# Antibiofilm activity of biosynthesized silver and copper nanoparticles using *Streptomyces* S29

**DOI:** 10.1186/s13568-023-01647-3

**Published:** 2023-12-06

**Authors:** Soha Elshaer, Mona I. Shaaban

**Affiliations:** Department of Microbiology and Immunology, Faculty of Pharmacy, Mansoura University, Mansoura, 35516 Egypt

**Keywords:** Antibiofilm, Silver nanoparticles, Copper nanoparticles, *Streptomyces*, Biologically synthesized nanoparticles, Elimination of mature biofilm

## Abstract

Microbial resistance and biofilm formation have been considered as the main problems associated with microbial resistance. Several antimicrobial agents cannot penetrate biofilm layers and cannot eradicate microbial infection. Therefore, the aim of this study is the biological synthesis of silver and copper nanoparticles to assess their activities on bacterial attachment and on the viability of dormant cells within the biofilm matrix. Ag-NPs and Cu-NPs were biosynthesized using *Streptomyces* isolate S29. The biologically synthesized Ag-NPs and Cu-NPs exhibited brown and blue colors and were detected by UV/Vis spectrophotometry at 476 and 594 nm, respectively. The Ag-NPs showed an average size of 10–20 nm as indicated by TEM, and 25–35 nm for Cu-NPs. Both Ag-NPs and Cu-NPs were monodispersed with a polydispersity index of 0.1–0.546 and zeta potential were − 29.7, and − 33.7 mv, respectively. The biologically synthesized Ag-NPs and Cu-NPs significantly eliminated bacterial attachment and decreased the viable cells in the biofilm matrix as detected by using crystal violet and tri-phenyl tetrazolium chloride assays. Furthermore, Ag-NPs and Cu-NPs significantly eradicated mature biofilms developed by various Gram-negative pathogens, including *A. baumannii*, *K. pneumoniae* and *P. aeruginosa* standard strains and clinical isolates. Data were also confirmed at the molecular level with prominent elimination of biofilm gene expression *carO, bssS* and *pelA* in *A. baumannii*, *K. pneumoniae* and *P. aeruginosa,* respectively compared to untreated cells under the same conditions. As indicated, Ag-NPs and Cu-NPs could be used as adjuvant therapy in eradication of antibiotic resistance and biofilm matrix associated with Gram-negative bacterial infection.

**Graphical Abstract:**

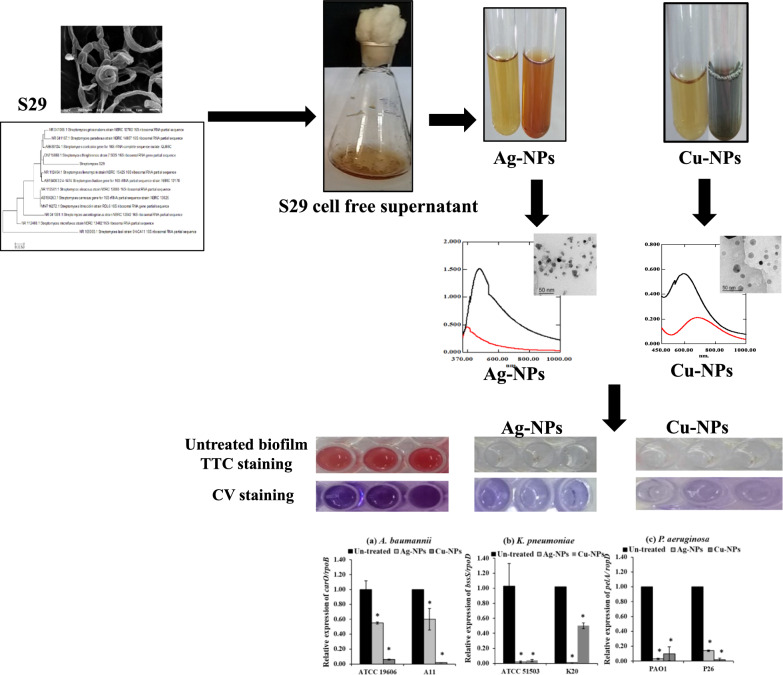

**Supplementary Information:**

The online version contains supplementary material available at 10.1186/s13568-023-01647-3.

## Introduction

Antibiotic resistance is one of the most dangerous and threatening problems for global health. The extensive use of antibiotics weakened their ability to eliminate bacteria and led to the spread of multidrug resistant (MDR) and extensively drug-resistant (XDR) Gram-negative pathogens. Expanding microbial resistance to carbapenems, fosfomycin, and colistin, the last resort antimicrobial regimens, has been described worldwide, which increases the rate of morbidity and mortality associated with the development of fatal pathogens (Aslam et al. 2018). Hence, we need to explore new strategies to combat MDR isolates (Morris et al. 2019). Furthermore, the rapid development of resistance to any newly developed antimicrobial agent halted pharmaceutical companies’ investment in antimicrobial research and industry, as once an antimicrobial agent is released, bacterial resistance develops within a short period with low and curtailed profits (Ventola 2015).

In addition, Gram-negative bacteria retain the ability to survive in the host through the formation of biofilm. Biofilm is protective layers composed of polysaccharides, proteins and eDNA that support host colonization and evasion. Inside the biofilm, bacterial cells remain dormant, and protected from the immune system. The accumulation of the polysaccharide biofilm matrix develops a protective layer surrounding the pathogenic organisms and eliminates antimicrobial penetrations. The inner cells are more resistant and persistent to antimicrobial agents. Therefore, biofilm has a distinct role in the pathogenesis of microbial infection as a breeding medium for continual microbial growth (Vestby et al. 2020).

Recent developments in nano-antimicrobials revealed new strategies to combat MDR isolates and associated microbial virulence (Ozdal and Gurkok 2022). Metallic nanoparticles were developed as a new approach in the fields of medicine and industry. More nanometals and nano-oxides have attained distinguished antimicrobial effects, including zinc oxide (ZnO), silver (Ag), gold (Au), aluminum (AL), aluminum oxide (Al_2_O_3_) copper oxide (CuO), and selenium (Se). Additionally, metal and metal oxide nanoparticles have demonstrated improved durability, better selectivity, greater stability in aqueous media, minimal cost and more environmentally favorable characteristics than organic nanoparticles (Durán et al. 2016; Patil and Kim 2017). These properties support the interaction with the microbial cell wall, causing cellular damage and intracellular distribution. Biologically synthesized nanoparticles using bacteria, plants, algae, and fungi have received superior attention for research as these methods provide a safe and efficient strategies for the reduction and capping of salts into corresponding nanometals (Alsaiari et al. 2023). Microbial cells are characterized by ease of their mass cultivation, higher growth rate in addition to biomass production and their amenability of genetic manipulation, giving the ability of in vivo tuning nanoparticles properties. During the synthesis process, biological method prevents particle aggregation and provides plenty of proteins, amino acids and idiolites used as reducing agents. Additionally, microbial-based approach produces nanometals with defined morphology, size, monodispersity and chemical composition. *Streptomyces* is a filamentous Gram-positive bacterial genus, known for safety and human non-pathogenicity, producing various secondary metabolites, pollution free and environment friendlier. So among *Actinomycetes*, *Streptomyces* is the most candidates in the field of nanotechnology for the biological synthesis of Ag- and Cu-NPs on large scale and at low cost (Eid et al. 2020; Prasad et al. 2016).

Nanomaterials loaded with antibiotics act through different pathways and mechanisms that enable them to combat microbial resistance and to fulfil the shortcomings of antibiotics. However, less is known about the effect of nanometals on bacterial attachment and their effect on biofilm formation.

Therefore, the aim of this research is the green synthesis of Ag-NPs and Cu-NPs using *Streptomyces* isolated from soil as an efficient and safe method for the biosynthesis of nanometals. The bioformed nanoparticles were characterized by analysis of particle size and zeta potential, UV spectrophotometry, transmission electron microscopy (TEM), selected area electron diffraction pattern (SAED) analysis and energy dispersive X-ray diffraction (EDX). Then, the influence of the synthesized nanoparticles on microbial adhesion and mature biofilm formation in Gram-negative bacteria was assessed. The effect of the biogenic Ag-NPs and Cu-NPs on the expression of biofilm regulatory genes was also evaluated.

## Materials and methods

### Bacterial isolates

Gram-negative bacterial standard strains, *A. baumannii* (ATCC 19606), *K. pneumoniae* (ATCC 33495 and ATCC 51503), and *P. aeruginosa* PAO1 were utilized in the present study. Clinical isolates of *Acinetobacter*, *Klebsiella,* and *Pseudomonas* were collected from different sources, Mansoura University hospitals, after the ethical approval of the research ethical committee, Faculty of Pharmacy, Mansoura University in compliance with the requirements of medical research in the handling and usage of human subjects (Jama 2013). Identification of the isolates was carried out based on microbiology standard techniques (Collee et al. 1996; Holt et al. 1994; MacFaddin 2000). All purified isolates were subjected to cryo-storage at − 80 °C as glycerol stock (30%) and deposited at the Microbial Resistance Culture Collection MRCC registered as WDCM1274 under the name *Streptomyces* MRCC S29.

### Media and chemicals

ISP2 media were used for the cultivation of *Streptomy*ces isolates (pH 7.2) (glucose 4 gm, yeast extract 4 gm and malt extract 10 gm), and for solid ISP2 plates nutrient agar 20 gm/L was added. Silver nitrate (AgNO3), and copper sulfate (CuSO4) were all purchased with 99.9% purity from Sigma Aldrich in the United Kingdom.

### Isolation, purification, and microscopic examination of environmental samples

Different soil samples were collected by digging 10–20 cm below the ground, and 5 g of the soil was collected in sterile containers. The collected samples were dried at 50 ℃ for 10 min. One gram of the dried soil was diluted with 10 mL of sterile NaCl (0.9% v/v) with subsequent mixing for 15 min. The obtained soil suspension was diluted 1/10 in sterile saline solution with good mixing. Diluted samples were streaked on ISP2 plates and incubated at 28–30 ℃ for 7–10 days. Round, chalky, hard, and colored colonies were subsequently purified in new ISP2 plate. Simple and Gram-staining of the purified colonies were performed and examined under a microscope.

### Selection of *Streptomyces* isolates for biosynthesis of nanometals

The metal salts, silver nitrate (AgNO3), and copper sulfate (CuSO4) at different concentrations (1–10 mM) were added to ISP2 media to prepare metal supplemented ISP2 plates. The pure *Streptomyces* isolates were propagated on these metal supplemented ISP2 plates and incubated at 28–30 °C for 7 days. *Streptomyces* isolates that can grow on plates supplemented with high salt concentrations were assigned and selected for the synthesis of nanometals (Shaaban and El-Mahdy 2018).

### Optimization of different parameters for production of NPs by *Streptomyces* S29

#### Nature and concentration of copper salt

Cell-free supernatant (15 ml) was added to different concentrations of CuSO4 (3 and 5 mM) and CuCl2 (5mM). The blend was incubated in the dark under continuous agitation at 28–30 **°**C and UV–Vis spectroscopy was used to evaluate the formed Cu-NPs**.**

#### Incubation time

Each metal salt AgNO3 and CuSO4 at fixed concentration (5 mM) were added to 15 ml of S29 filtrate and both mixtures were incubated as above. Creation of Ag- and Cu-NPs was analyzed by UV–Vis spectra per 24 h intervals up to 3 days.

### Microbial synthesis of metallic nanoparticles

The *Streptomyces* isolate S29 that can survive at high salt concentrations was selected and propagated on ISP2 plates for 5–10 days at 28–30 °C. Bacterial spores were scraped from the surface of the ISP2 plates, and the spore suspension was prepared in tween water (0.1 v/w). The prepared *Streptomyces* spores were inoculated in ISP2 media (50 mL) at a final concentration of 1 × 10^6^ CFU/mL, and the media were cultivated at 28–30 °C, for 5–7 days with shaking.

Bacterial cells were harvested by centrifugation at 6000 xg for 15 min at 4 °C and the cell-free supernatant was used to produce metallic nanoparticles. The cell-free supernatant (pH = 8–8.5) was collected and mixed with metallic salts; 5 mM from each AgNO3 and CuSO4, for the synthesis of silver and copper nanoparticles, respectively. In addition, cell-free supernatants without added metallic salts AgNO3 and CuSO4 were also included. All samples were kept in shaker at 28–30 °C in darkness up to 3 days and the cell-free supernatants treated with the corresponding metals were collected after 24 and 48 h, and the biologically formed nanoparticles were characterized by various techniques (Chauhan et al. 2013).

### Characterization of the biosynthesized nanoparticles

#### Color development and UV–Visible spectroscopy

The reduction of metallic ions by *Streptomyces* extract and the biological synthesis of nanometals were primarily observed by monitoring the color change of the treated *Streptomyces* supernatant. Additionally, the absorption spectra (**λ**_**max**_) in the wavelength range of 300–1000 nm were detected at a resolution of 1 nm using UV/Vis spectrophotometer 1601 pc (Saminathan 2015) using untreated cell-free supernatant as blank. Additionally, the UV/Vis spectra of the supernatant were performed using ISP2 media as blank.

#### Determination of particle size and zeta potential

A particle size analyzer (Microtrac, nanotrac wave II Q) was used to detect the particle size, zeta potential and polydispersity index of the biosynthesized nanoparticles. The principle of the detection comprises the use of the dynamic light scattering technique of laser light for assessment of the particle size and particle size distribution of the formed nanoparticles and their associated charge (Ashizawa 2019).

#### Scanning electron microscopy

The topography and surface characteristics of the biologically prepared metallic nanoparticles were inspected by scanning electron microscopy (SEM) (JEOL JSM 6510 lv, JEOL Ltd, Tokyo, Japan) (Vladár and Hodoroaba 2020).

### TEM and SAED analysis

The morphological properties of the formed metallic nanoparticles were also characterized using transmission electron microscopy (TEM) (JEOL JEM-2100). A carbon coated copper grid was loaded with the tested samples, and the presence of Ag-NPs and Cu-NPs was examined with TEM (power X 1.000–800.000 (JEOL JEM-2100). SAED was also detected for the same samples (Ag-NPs and Cu-NPs) examined by TEM.

### EDX Analysis

The elemental compositions of Ag-NPs and Cu-NPs were detected by EDX microanalytical system operated with a scanning electron microscope (SEM) (JEOL JSM 6510 lv, JEOL, Pleasanton, CA, USA). The glass cover slips were coated with thin film of Ag-NPs and Cu-NPs, sprayed with gold–palladium and examined using a Jeol JSM-6510 L.V SEM at an accelerating voltage of 30 kV (Pleasanton, CA, USA).

### FTIR spectroscopy

Ag-NP and Cu-NP samples were prepared for FTIR by centrifugation at 6000 xg for 30 min. The pellets were washed, resuspended in deionized water, and freeze dried. Potassium bromide was added to the lyophilized Ag-NPs and Cu-NPs and examined using a fully integrated Thermo-Nicolet 6700 FTIR spectrophotometer (Thermo Scientific, Waltham, MA, USA) and provided with ATR. The FTIR spectrum was scanned at 400–4000 cm^−1^ using a resolution of 4 cm^−1^. The spectra were represented as percentage transmittance on the x-axis and the wavelength (cm^−1^) on the y-axis.

### Strain identification

*Streptomyces* isolates that can survive and grow on ISP2 plates supplemented with AgNO3, and CuSO4 were selected and identified via the construction of the neighbor-joining tree. Bacterial cells were harvested in sterile RNase and DNase-free H_2_O, and boiled for 10 min at 95 °C, and the cell lysate was centrifuged, separated in new tubes, and used as a template for amplification of 16S rRNA gene.

PCR amplicon of 16S rRNA gene was obtained using 16S rRNA for (AGAGTTTGATCCTGGCTCAG) and 16S rRNA rev (AGAAAGGAGGTGATCCAGCC). The reaction mixture was composed of 12.5 µL of Dream Taq Green PCR Master Mix (Fermentas, USA), 0.5 µL of primer pairs of each primer (10 µM) and 1 µL of bacterial lysate in a final volume of 25 µL PCR mixtures. Simultaneously, a PCR mixture without DNA template was run as a PCR negative control. The PCR conditions for amplification of 16S rRNA gene involved an initial cycle of denaturation for 3 min at 95 °C, followed by 35 cycles beginning with denaturation at 95 °C for 30 s, primer annealing at 47 °C for 30 s, and extension at 72 °C for 90 s, and ending with a final extension cycle at 72 °C for 10 min. The PCR amplicons were run on agarose gel (1.5% w/v) and purified using Qiagen gel purification kits (Qiagen, USA) according to the manufacturers’ instructions and sequenced (Tamura et al. 2011). The obtained sequence was subjected to a BLAST search against NCBI with high similarity variants. For strain identification, the phylogenetic tree was constructed using MEGA5 software (v. 7.2) based on the neighbor binding method (Tamura et al. 2011) using *Nocardiopsis alborubidus* DSM 40465 as an outgroup strain.

Also, *Streptomyces* isolate S29 was cultivated on ISP2 media for 4–5 days until sporulation. Cups of the spores (1 mm in diameter) were cut from the media and stained for visualization using scanning electron microscopy (SEM) with magnification power 5000–10.000 X (JEOL JSM 6510 lv, JEOL Ltd, Tokyo, Japan).

### Antimicrobial activity of the synthesized nanometals

The minimal inhibitory concentration (MIC) of the biologically synthesized nanoparticles was determined using the broth microdilution method (CLSI 2020). Also, the antimicrobial activity of the *Streptomyces* S29 cell-free supernatant was performed and ISP2 media only was included in each plate as a negative control. Muller Hinton broth (100 µL) was distributed in the wells of sterile microtiter plates. The prepared nanometals were diluted 1:1 in the wells. Each well received the diluted culture until the final inoculum size was 1 × 10^5^ CFU/well. The plates were incubated at 37 °C overnight. The MIC of each nanometal was calculated as the lowest concentration that inhibited microbial growth. As well, the minimal bactericidal concentration (MBC) of each nanopreparation was calculated as the lowest concentration that killed the microbial growth.

### Antibiofilm activities of biosynthesized nanoparticles against Gram-negative isolates

#### Inhibition of bacterial adherence

The effect of the biosynthesized nanoparticles on bacterial attachment, and initiation of biofilm formation was detected. Both standard and selected clinical isolates were grown in tryptic soy broth (TSB) at 37 °C for 18 h, and bacterial cultures were diluted until they reached 0.5 McFarland turbidity. Each nanoparticle at final concentrations of 0.25x, 0.5x, 1x, and 2x  MIC was distributed in the wells of 96-well flat-bottomed microplates. The wells were inoculated with the diluted bacterial cultures. Wells without nanoparticles, receiving TSB (pH 7.3–7.5) only, were also included as a negative control in each plate. The plates were incubated at 37 °C for 24 h (Yu et al. 2018).

The biomass and metabolic activity of the biofilm formed by each of the tested isolates were quantified by the crystal violet (CV) and 2, 3, 5 tri-phenyl tetrazolium chloride (TTC) assays, as represented below. The minimum biofilm inhibitory concentration (MBIC_50_) was calculated for each of the prepared metallic nanoparticles. MBIC_50_ was specified as the lowest concentration of nanopreparation that inhibits the formation of biofilm by 50%.

#### Biofilm eradication assay

The effect of bioformed nanoparticles on the preformed biofilm of all tested isolates was also assessed. Briefly, 24 h-old cultures of the strains were diluted in fresh TSB at a final cell concentration of 1.5 × 10^8^ CFU/mL. The tested isolates were inoculated (200 µL) in each well of a sterile 96-well flat-bottom sterile polystyrene microtiter plate. TSB was added to some wells to serve as a negative control. The plates were incubated at 37 °C for 24 h to allow complete maturation of biofilm. Afterwards, the culture supernatants were carefully discarded, and all wells were rinsed twice with sterile saline. Fresh TSB (200 μL) was added to the negative control wells, and TSB was challenged with 0.25x, 0.5x, 1x, and 2x  MICs of Ag-NPs and 25%, and 50% of Cu-NPs were added to the corresponding wells. The plates were incubated at 37 °C for 24 h and the effect of nanoparticles was determined with the CV and TTC assays as described below (Yu et al. 2018). The minimum biofilm reduction concentration (MBRC_50_) was calculated for each treated nanoparticle concentration. MBRC_50_ was calculated as the lowest concentration of the nanometal required to disrupt at least 50% of the preformed biofilm.

#### Quantification of biofilm with crystal violet and tri-phenyl tetrazolium chloride

The biomass of the tested bacterial biofilm and their metabolic activity were evaluated utilizing CV and TTC assays (Mishra et al. 2017). After the tested plates were incubated, the planktonic bacterial cells were aspirated, and the wells were washed twice with sterile saline to remove the remaining unattached cells.

In the CV method, 250 μL of methanol was added for fixation of the formed biofilm, and methanol was maintained in the wells for 20 min. Then, excess methanol was removed, and the plates were allowed to dry. Finally, the formed biofilm was stained with 1% w/v CV for 20 min. The unadhered dye was rinsed, and 250 μL of 33% (v/v) glacial acetic acid was applied to dissolve the formed biofilms.

For the TTC assay, the viability of cells was detected by adding 200 μL of TTC (10 mg/mL) to each well, and the plates were incubated at 37 °C until the appearance of a red color in untreated wells. In both assays, the plates were measured at OD 570 nm using ELISA plate reader. The percentage inhibition of biofilm formation by Ag- and Cu-NPs was calculated in comparison with untreated isolates.

### RNA extraction and RT-PCR analysis

To study the effect of biosynthesized Ag-NPs and Cu-NPs on the expression of biofilm genes, the standard isolates *A. baumannii* (ATCC 19606), *K. pneumoniae* (ATCC 51503), and *P. aeruginosa* PAO1 were grown in the presence and absence of sub-MICs of Ag-NPs and Cu-NPs. The cells with and without the tested nanoparticles were grown to the end of the exponential stage (OD630 of 0.5–0.6 nm) and the cells were harvested by centrifugation at 8000 xg for 30 min at 4 °C. The supernatant was discarded, and the pellets were dried and treated with triazole reagent (Sigma Aldrich, UK). The cells were homogenized, and total RNA was extracted according to the triazole RNA extraction protocol.

For analysis of gene expression, genomic DNA was removed using gDNA wipeout buffer and cDNA synthesis was performed using Quantiscript reverse transcriptase enzyme provided with the QuantiTech Reverse transcription kit (Qiagen, USA).

RT-PCR was performed using SYBR Green, no ROX according to the manufacturer’s instructions (Enzynomics, Korea) on Rotor-gene Q (Qiagen, USA) using biofilm specific primers (Additional file 1: Table S1). The expression of each gene was normalized to that of the housekeeping genes, *rpoB*, *rpoD*, and *rpoD* for *A. baumannii K. pneumoniae* and* P. aeruginosa*, respectively. The data were calculated as relative expression using the 2^−ΔΔCt^ method as previously described (Pfaffl 2001) and the fold change in the expression of the cells treated with either Ag-NPs or Cu-NPs was also calculated and related to the expression of the control untreated cells.

### Statistical analysis

The data are represented as the mean ± standard deviation using an Excel spreadsheet. GraphPad Prism software (version 5.01) was used for statistical analysis of the data. The mean of the treated samples was compared with the mean of untreated control groups propagated under the same conditions using chi-square test, and a *P* value < 0.05 was considered statistically significant.

## Results

### Selection of *Streptomyces* isolates for biosynthesis of nanometals

*Streptomyces* isolate S29 grew on plates supplemented AgNO3, and copper sulfate CuSO4 up to 10 mM were assigned and selected for the synthesis of Ag and Cu nanometals (Shaaban and El-Mahdy 2018).

### Optimization of metallic nanoparticles formation

Upon utilization of CuSO4 (3 mM) by *Streptomyces* S29, it revealed low Cu-NPs yield since the λ_max_ at 622 nm was 0.263; while at concentration of 5 mM, it gave λ_max_ of 0.565 at 594 nm (Additional file 1: Fig. S1a). Further, the measured absorbance of cell-free supernatant challenged with 5 mM of CuCl2 (λ_max_ of 0.565 at 594 nm) was slightly lower than that challenged with 5 mM of CuSO4 but with no significant difference (Additional file 1: Fig. S1b). Unfortunately, CuCl2-based nanopreparation has unacceptable large particle size of 508.1 nm with PDI of 0.419 and zeta potential of − 28.8 mv (Additional file 1: Fig. S1c).

The UV–Vis spectra exhibited an increase in peak intensity of Ag-NPs after 24 h more than 48 h incubation time (λ_max_ = 1.517 vs 1.4 at 478 nm, respectively.  (Additional file 1: Fig. S1d, Table S2). Inversely, Cu-NPs recorded higher absorbance reading after 48 h (λ_max_ = 0.565 at 594 nm) than 24 h (λ_max_ = 0.52 at 627 nm,  Additional file 1: Fig. S1e, Table S2). After the third day, there was no change in peak intensity, indicating that the metal bio-reduction and nano-formation was completed.

### Characterization of the bioformed nanoparticles

#### Color change and UV/Vis spectral analysis

**Fig. 1:**
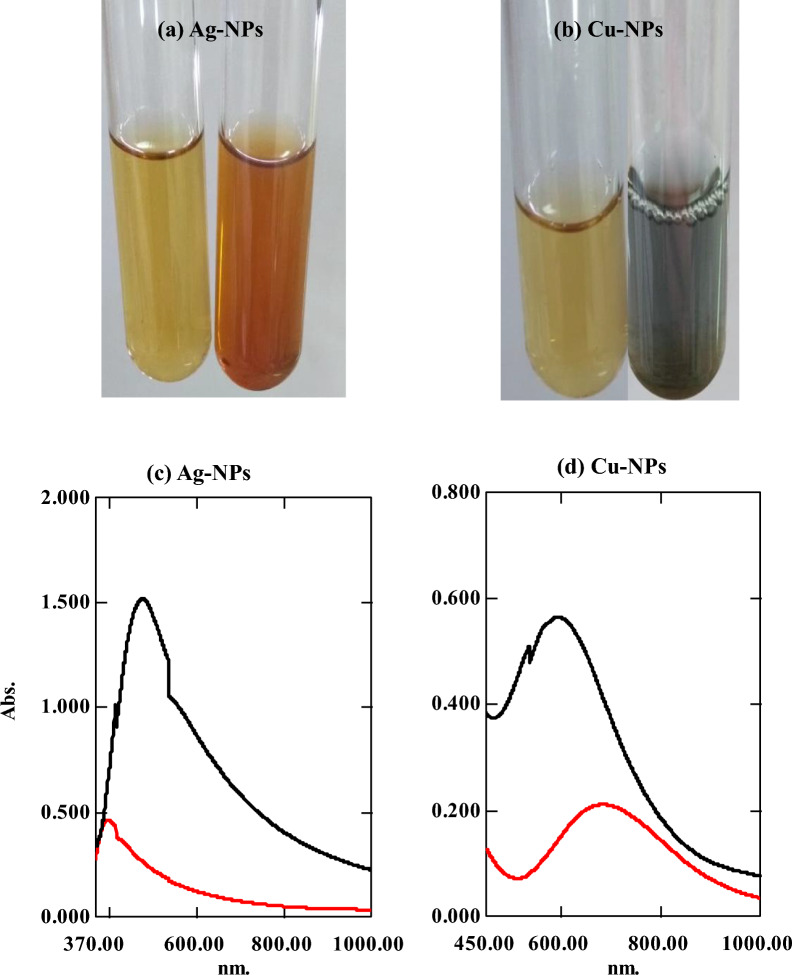
Distinct colors and UV/Vis spectrum of the bioformed nanoparticles using cell-free supernatant of *Streptomyces* isolate S29 (**a**) synthesized Ag-NPs with brown color, (**b**) Cu-NPs with greenish blue color (**c**) UV/Vis spectrum of Ag-NPs, and (**d**) UV/Vis spectrum of Cu-NPs (black curve: cell-free filtrate added salts, red curve: cell-free filtrate only)

The formation of metallic nanoparticles was assessed by visual observation of the color change of the treated supernatant within 24–48 h after adding salts. The color of the treated supernatant with AgNO3 changed to brown, indicating the formation of Ag-NPs (Fig. 1a). This was confirmed by the presence of surface plasmon resonance of Ag-NPs at 478 nm (Fig. 1c) as detected by UV/Vis spectrophotometry (Chauhan et al. 2013; Soltaninezhad et al. 2015).

The color of CuSO4 changed from green to blue or bluish green, which indicates the formation of Cu-NPs (Fig. 1b). The color change is due to the surface plasma resonance associated with the biosynthesis of Cu-NPs in the filtrate containing metabolites with a maximum absorption peak at 594 nm (Fig. 1d).

#### Determination of polydispersity index and zeta potential

The polydispersity index (PDI) was in the range of 0.1- 0.546. The diameters of Ag, and Cu-NPs were 158, and 190.3 nm, respectively, as determined by the light scattering method (Additional file 1: Figure S2a, b). The zeta potentials of the biosynthesized nanoparticles indicate the charge acquired by the surface of the formed nanoparticles. The zeta potentials of biosynthesized Ag-NPs, and Cu-NPs were, -29.7, and -33.7 mv respectively (Additional file 1: Figure. S2c, d).

#### Characterizations of nano-silver and copper preparations

**Fig. 2:**
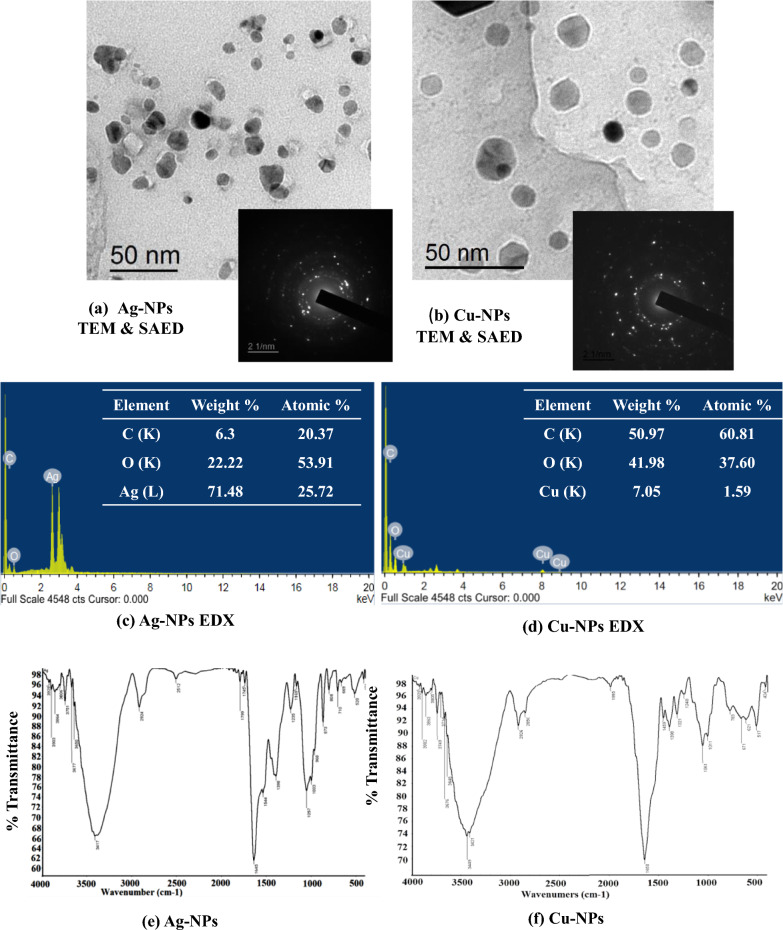
Characterization of NPs. Transmission electron microscopy (TEM) image and selected area electron diffraction pattern (SAED) analysis of (a) Ag-NPs, and (b) Cu-NPs. Energy dispersive X-ray diffraction (EDX) of (c) Ag-NPs, and (d) Cu-NPs. FTIR spectra of biosynthesized (e) Ag-NPs and (f) Cu-NPs using extracellular extract of *Streptomyces* S29

Transmission electron microscopy (TEM) analysis was performed to characterize the morphological shape and size of the biosynthesized NPs. The biosynthesized Ag-NPs obtained by utilizing the cell supernatant of *Streptomyces* S29 were spherical with a uniform crystalline nature and an average size of 10 to 20 nm (Fig. 2a).

Similarly, the biosynthesized Cu-NPs had a monodispersed diverse morphological shape ranging from spherical to square and hexagonal with a dimensional size of 25–35 nm (Fig. 2b).

SAED analysis was also performed to characterize the crystal structure of the prepared Ag-NPs and Cu-NPs. The SAED analysis indicated the spherical crystalline structure of Ag-NPs (Fig. 2a), while it showed that Cu-NPs were amorphous due to the diffused ring patterns (Fig. 2b).

EDX analysis was used to detect the element formation of Ag-NPs and Cu-NPs. The characteristic signals of silver atoms were identified in the energy range of 2.5 keV, indicating the elemental synthesis of Ag-NPs. Additionally; other signal peaks at 3–3.5 keV indicate the binding energy of crystalline metallic Ag (Fig. 2c). Both C and O atoms were also detected with Ag**;** however, Ag represents the highest atomic weight and percentage. EDX data of the biogenic Cu-NPs indicated the absorption peaks of copper at 1, 8 and 9 keV with oxygen as the main element composition (Fig. 2d).

FTIR spectra of Ag-NPs and Cu-NPs synthesized from S29 solutions were detected (Fig. 2). The Ag-NP spectra show characteristics broadening bands recognized at 1057, 1235, 1398, 1544, 1645 1745, 1799, 2924 and 3417 cm^−1^ absorption peaks. The band at 1057 cm^−1^ was assigned to the (amine) C–N stretching vibration of the proteins. The band at 1235 cm^−1^ corresponds to the C–N stretching of amines. The presence of bands at 1398 and at 1544 cm^−1^ indicated C– OH stretching vibrations of the alcoholic group and –C–O group, respectively. The band at 1645 was detected in the spectra corresponding to C–C stretching. The appearance of bands at 1745 and 1799 cm^−1^ was detected with non-conjugated C–C stretching. The band at 2924 indicated the region arising from C–H stretching of aromatic compound. The band at 3417 cm^−1^ indicated the presence of alcohol and phenol (Fig. 2e).

Concerning Cu-NPs, (Fig. 2f) showed spectral peaks at 517, 671, 1061, 1248, 1398, 1459, 1650, 1995, 2856, 2936, 3449, and 3749 cm − 1. Bands at 1061 and 1248 revealed C = C stretching of the aromatic ring and C–O stretching of the ester, respectively. Bands at 1459 and 1650 cm^−1^ indicated asymmetric stretching COO − . The presence of bands at 2936 and 2856 cm^−1^ is due to stretching of aliphatic groups. The broad bands at 3449 and 3749 cm − 1 elicited the stretching of -OH groups.

### Characterization of *Streptomyces* isolate S29

#### Molecular analysis and microscopic characterization of *Streptomyces* isolate S29

**Fig. 3:**
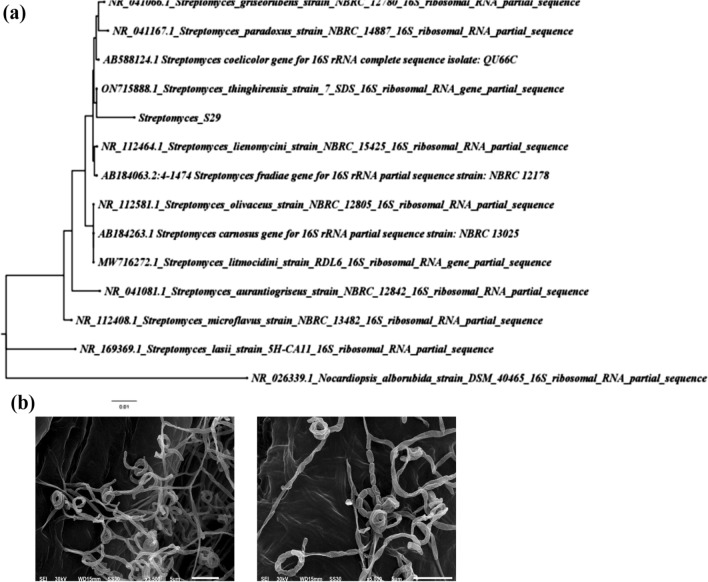
Characterization of *Streptomyces* isolate S29; (a) Phylogenetic tree of *Streptomyces* S29 based on sequence alignment of 16S rRNA gene of isolate S29 and other *Streptomyces* species. The phylogenetic tree was constructed using the neighbor-joining method of MEGA 7.0 (b) SEM figures of *Streptomyces* isolate S29 indicating spiral spore chains with smooth surfaces

Sequencing of the 16S rRNA gene of the *Streptomyces* S29 isolate was performed. BLAST search analysis of NCBI and the obtained sequences were analyzed using the neighbor-joining method using alignment and phylogeny tools of MEGA 0.7 software, which revealed that isolate S29 was *Streptomyces sp*. A dendrogram was constructed, which indicated that isolate S29 showed high similarity to *Streptomyces thinghirensis* with 98.82% (Fig. 3a). The obtained sequence was submitted to GenBank (NCBI) under an accession number OQ726419.

SEM examination of S29 showed that spore chains were spiral with a smooth surface (Fig. 3b) with similar morphological characteristics to *Streptomyces thinghirensis* isolated from Thinghir*,* Morocco (Loqman et al. 2022).

### Antimicrobial activity and antibiofilm activity of Ag-NPs and Cu-NPs

#### Antimicrobial activity of Ag-NPs and Cu-NPs

The present study examined the antimicrobial activity of the biologically prepared nanoparticles, including ampicillin/sulbactam and ceftazidime (each at 16384 µg/ml concentration) positive antibiotic controls (Table 1). Ag-NPs exhibited prominent antimicrobial activity against all tested isolates, with MICs of 2.1–4.2 µg/mL against *A. baumannii* and *P. aeruginosa*. Additionally, the biogenic Ag-NPs were effective against *K. pneumoniae* with an MIC range of 4.2–8.4 µg/mL*.* In addition, the prepared Ag-NPs exhibited bactericidal activity against all the tested isolates in a range from 2.1 to 33.7 µg/mL.

**Table 1:** The antibacterial of Ag-NPs and Cu-NPs

Bacterial isolates	Ag-NPs (539.4 µg/ml)		Cu-NPs (317.73 µg/ ml)		Ampicillin/Sulbactam (µg/ml)		Ceftazidime (µg/ml)	
	MIC (µg/mL)	MBC (µg/mL)	MIC (µg/mL)	MBC (µg/mL)	MIC (µg/mL)	MBC (µg/mL)	MIC (µg/mL)	MBC (µg/mL)
*A. baumannii* ATCC 19606	4.2	4.2	> 158.87	> 158.87	256	256	< 4	< 4
A10	4.2	16.86	> 158.87	> 158.87	1024	1024	2048	2048
A11	2.1	2.1	> 158.87	> 158.87	128	128	512	512
*K. pneumoniae* ATCC 33495	8.43	8.43	> 158.87	> 158.87	8192	8192	8192	8192
*K. pneumoniae* ATCC K51503	8.43	8.43	> 158.87	> 158.87	128	128	32	32
K7	8.43	8.43	> 158.87	> 158.87	> 8192	> 8192	8192	8192
K20	4.2	4.2	> 158.87	> 158.87	> 8192	> 8192	8192	8192
*P. aeruginosa* PAO1	2.1	2.1	> 158.87	> 158.87	256	512	< 4	< 4
P2	4.2	33.7	> 158.87	> 158.87	> 8192	> 8192	4096	4096
P26	4.2	4.2	> 158.87	> 158.87	512	512	< 4	64

In contrast, the biosynthesized Cu-NPs did not reveal any antimicrobial effect against standard and clinical isolates of *A. baumannii, K. pneumonia*, and *P. aeruginosa.* The results showed that the MIC and MBC of Cu-NPs were > 158.87 µg/ml (Table 1). Also, the cell-free supernatant of *Streptomyces* S29 did not show any antimicrobial effect against the tested isolates.

### Effect on bacterial adherence

**Fig. 4:**
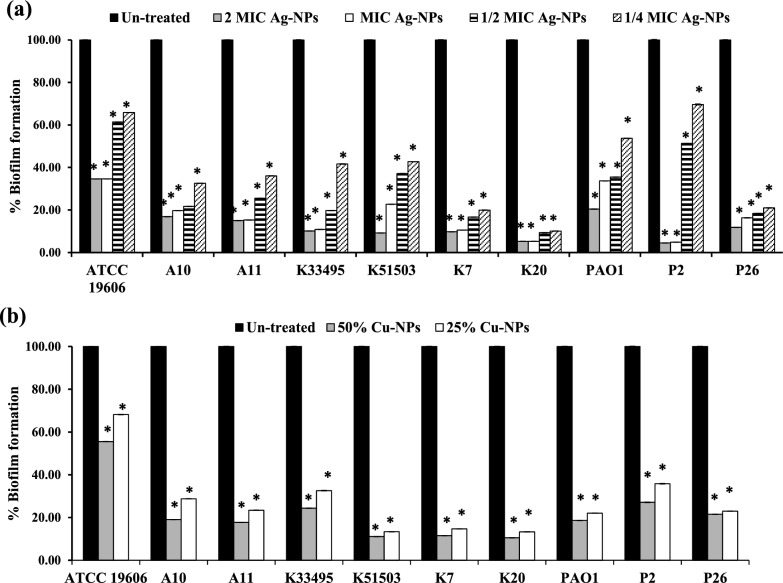
Effect of different concentrations of (**a**) Ag-NPs and (**b**) Cu-NPs on the initial attachment and biofilm formation by *A. baumannii* ATCC 19606, *K. pneumoniae* ATCC 33495, *K. pneumoniae* ATCC 51503, *P. aeruginosa* PAO1 standard strains and *A. baumannii* (A10, A11), *K. pneumoniae* (K7, K20) and *P. aeruginosa* (P2, P26) clinical isolates. The biofilm was stained with CV. Values represent the mean ± SD of three independent experiments (*, significant, *P* < 0.001)

The initial attachment and biofilm-forming ability of all the tested standard and clinical isolates were measured in the presence and absence of different concentrations of nano-biosynthesized particles using the microtiter plate assay. The results demonstrated a significant reduction in biofilm formation in treated isolates compared to untreated cells in a concentration dependent manner (*P* < 0.001, Fig. 4 and Additional file 1: Figs S3-5). In the CV assay, Ag-NPs at 2 × and 1 × MICs decreased the biofilm formation in the range of 65–95%, at 0.5x  MIC by 39–91% and at 0.25x  MIC by 30–90% (Fig. 4a)*.* Cu-NPs also reduced the initial bacterial adherence by 44–90% and by 32–87% at 50% and 25% of its concentration, respectively (Fig. 4b).

**Fig. 5:**
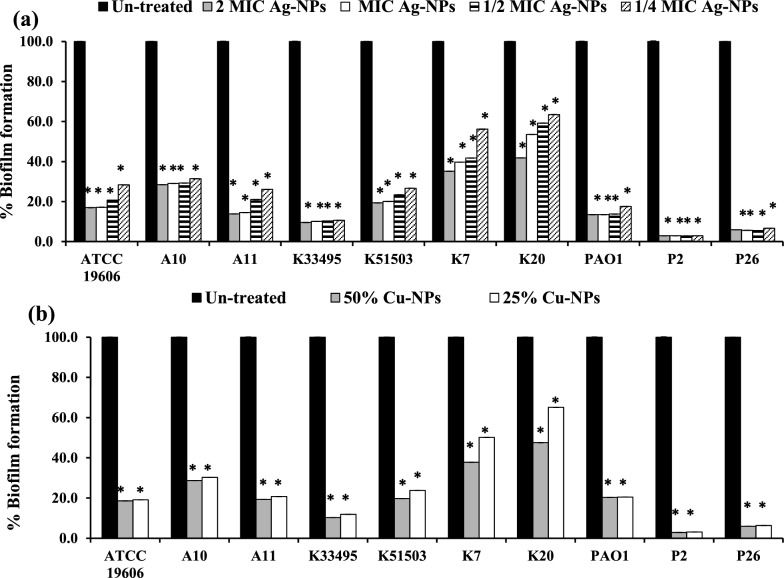
Tri-phenyl tetrazolium chloride (TTC) assay for studying the effect of (**a**) Ag-NPs at 0.25, 0.5, 1x  and 2x  MICs and (**b**) Cu-NPs at 25% and 50% on bacterial attachment and biofilm formation by *A. baumannii* ATCC 19606, *K. pneumoniae* ATCC 33495, *K. pneumoniae* ATCC 51503, *P. aeruginosa* PAO1 standard strains and *A. baumannii* (A10, A11), *K. pneumoniae* (K7, K20) and *P. aeruginosa* (P2, P26) clinical isolates. Values represent the mean ± SD of three independent experiments (*, significant, *P* < 0.001)

In the TTC assay, compared to the control groups, biofilm treated cultures with different doses of Ag-NPs and Cu-NPs significantly repressed the initial stage of biofilm formation, with prominent activity against *Acinetobacter* and *Pseudomonas* isolates. The highest inhibition of early-stage biofilm was observed in *P. aeruginosa* isolate P2 at 97%, while the least inhibition was observed in *K. pneumonia* isolate K20 at 58%, 46%, 41%, and 37%, in challenged cultures with 2x, 1x, 0.5x  and 0.25x  MIC Ag-NPs, respectively, (*P* < 0.001, Fig. 5a). Similarly, Cu-NPs exhibited a 52–97%, and 35–97% reduction in biofilm biomass at 50% and 25% of its concentration, respectively (*P* < 0.001, Fig. 5b and Additional file 1: Figs S3–S5).

Among all isolates, Ag-NPs were more potent than Cu-NPs in reducing the initial colonization for biofilm formation, since the MBIC_50_ of Ag-NPs ranged from 0.53–8.4 µg/ml while that of Cu-NPs ranged from 79.4 to > 158.87 µg/ml (Table 2).

**Table 2:** The antibiofilm activity of Ag-NPs and Cu-NPs

Compounds	Bacterial isolates	MBIC_50_ µg/ml		MBRC_50_ µg/ml	
		CV	TTC	CV	TTC
Ag-NPs (539.4 µg/mL)	*A. baumannii* ATCC 19606	4.2	1.05	1.05	1.05
	A10	1.05	1.05	1.05	1.05
	A11	0.53	0.53	16.8	0.53
	*K. pneumoniae* ATCC 33495	2.1	2.1	2.1	2.1
	*K. pneumoniae* ATCC K51503	2.1	2.1	2.1	2.1
	K7	2.1	4.2	2.1	2.1
	K20	1.05	8.4	16.8	8.4
	*P. aeruginosa* PAO1	1.05	0.53	4.2	2.1
	P2	4.2	1.05	8.4	2.1
	P26	1.05	1.05	2.1	> 67.2
Cu-NPs (317.73 µg/mL)	*A. baumannii* ATCC 19606	> 158.87	79.4	79.4	79.4
	A10	79.4	79.4	79.4	79.4
	A11	79.4	79.4	> 317.73	79.4
	*K. pneumoniae* ATCC 51503	79.4	79.4	317.73	79.4
	*K. pneumoniae* ATCC K51503	79.4	79.4	79.4	79.4
	K7	79.4	79.4	317.73	79.4
	K20	79.4	158.87	> 317.7	317.73
	*P. aeruginosa* PAO1	79.4	79.4	> 317.7	158.87
	P2	79.4	79.4	317.73	79.4
	P26	79.4	79.4	158.87	> 317.7

### Effect on mature biofilm

**Fig. 6:**
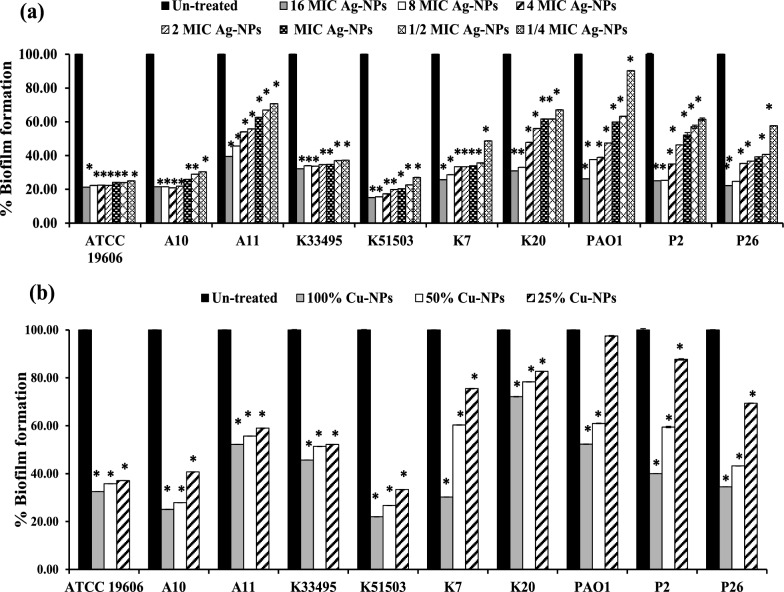
Effect of different concentrations of (**a**) Ag-NPs and (**b**) Cu-NPs on mature biofilm of *A. baumannii* ATCC 19606, *K. pneumoniae* ATCC 33495, *K. pneumoniae* ATCC 51503, *P. aeruginosa* PAO1 standard strains and *A. baumannii* (A10, A11), *K. pneumoniae* (K7, K20) and *P. aeruginosa* (P2, P26) clinical isolates. The biofilm was stained with CV. Values represent the mean ± SD of three independent experiments (*, significant, *P* < 0.001)

The effect of different concentrations of the biologically prepared Ag-NPs and Cu-NPs on the mature biofilm formed by the tested standard and clinical isolates was compared to the untreated mature biofilm using microtiter plates. Testing the effect of both nanometals on preformed bacterial biofilms by both CV and TTC assays showed robust perdition in biofilms formed by *Acintobacter* and *Klebsiella* more than in those formed by *Pseudomonas* isolates (*P* < 0.001, Fig. 6 and Additional file 1: Figs S6–S8). After the biofilm had grown for 24 h and was further stained with CV, the destruction rates of Ag-NPs concentrations ranging from 16 × MIC to 0.25 × MIC were approximately 10- 85% (Fig. 6a). Cu-NPs also reduced the mature bacterial biofilm by 28–78%, 22–73% and by 3–67% at 100%, 50% and 25% of its concentration, respectively (Fig. 6b).

**Fig. 7:**
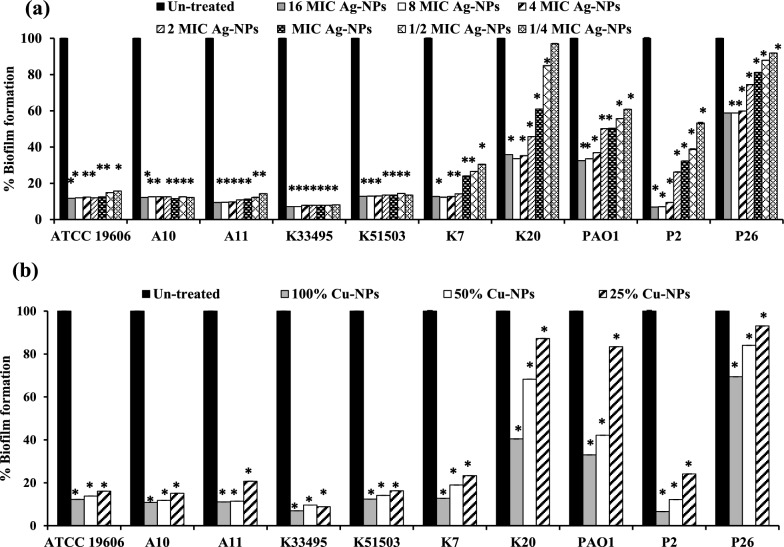
Tri-phenyl tetrazolium chloride (TTC) assay for studying the effect of (a) Ag-NPs at 0.25x, 0.5x, 1x, 2x, 4x, 8x and 16x  MICs and (**b**) Cu-NPs at 25%, 50% and 100% on mature biofilm of *A. baumannii* ATCC 19606, *K. pneumoniae* ATCC 51503, *K. pneumoniae* ATCC 51503, *P. aeruginosa* PAO1 standard strains and *A. baumannii* (A10, A11), *K. pneumoniae* (K7, K20) and *P. aeruginosa* (P2, P26) clinical isolates. Values represent the mean ± SD of three independent experiments (*, significant, *P* < 0.001)

In assays of cell viability using the TTC technique, the biofilm treated cultures with different concentrations of Ag-NPs and Cu-NPs disrupted the viable cells packed in the mature biofilm (Fig. 7 and Additional file 1: Figs S6-S8). In a concentration dependent manner, the biofilms treated with Ag-NPs were eliminated beginning from 3%, 12%, 19%, 25% and 40% to 92% at 0.25x, 0.5x, 1x, 2x and 4x  MIC, respectively, while the highest reduction rate (41%-93%) was achieved upon treatment with 8 × and 16 × MIC (*P* < 0.001, Fig. 7a).

Also, the reduction rates of 100% Cu-NPs on already-formed biofilms decreased to approximately 31–93% and 7–91% when diluted to 50% and 25%, respectively (*P* < 0.001, Fig. 7b, Table 2).

### Elimination of biofilm regulating genes

**Fig. 8:**
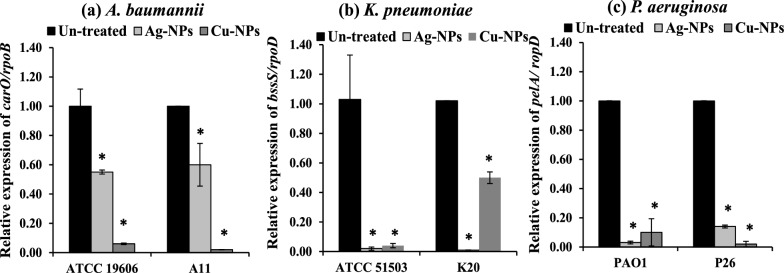
Fold change in the relative expression of (**a**) *carO/rpoB* in *A. baumannii* ATCC 19606 and A11, (**b**) *bssS/rpoD* in *K. pneumoniae* ATCC 51503 and K20 and (**c**) *pelA/ rpoD* in *P. aeruginosa* PAO1 and P26 treated with Ag-NPs and Cu-NPs compared to untreated cells. Values represent the mean ± SD of three independent experiments (*, significant, *P* < 0.00

The present study assessed the effect of Ag-NPs at 1/4  MIC on biofilm regulatory genes. Ag-NPs significantly (*P* < 0.001) eliminated the expression of *carO,* by 45%, and 40%, in the standard strain *A. baumannii* ATCC19060 and clinical isolate A11, respectively compared to their levels in the untreated cells (Fig. 8a). Furthermore, Ag-NPs significantly (*P* < 0.001) reduced the expression of the *bssS* biofilm regulatory gene in *K. pneumonia* ATCC51503 and K20 by 98 and 99%, respectively (Fig. 8b). Also, the level of expression of *pelA* in *P. aeruginosa* treated cells PAO1 and P26 cells treated with Ag-NPs was reduced by 97 and 86%, respectively (Fig. 8c).

Furthermore, Cu-NPs at 50% of their concentration significantly (*P* < 0.001) reduced the expression of *carO* in treated *A. baumannii* ATCC19606 and A11 by 94 and 98%, respectively (Fig. 8a). Cu-NPs also significantly (*P* < 0.001) eliminated the expression of *bssS* in treated *K. pneumonia* ATCC51503 and K20 by 98 and 99%, respectively (Fig. 8b). Moreover, the expression of *pelA* in *P. aeruginosa* treated cells PAO1 and P26 cells treated with Cu-NPs was eliminated by 90 and 98%, respectively (Fig. 8c).

## Discussion

The rate of microbial resistance is increasing rapidly. This led to prolonged hospital stay, and ineffective treatment of infectious diseases, with an increase in medical costs and elevation in mortality rates (Ventola 2015). In addition, biofilm-induced bacterial infection is a critical health menace. Microbial biofilms not only allow bacteria to cause diseases, but also aggravate the bacterial ability to colonize within the host. In the biofilm maturation stage, bacteria gain an inherent resistance to antimicrobial agents, providing a reservoir for systemic persistent infections. Biofilm-associated resistance is due to noticeable failure of antibiotics access into its robust exopolysaccharide matrix (Hall and Mah 2017).

The unique morphological, structural, chemical, mechanical, and optical characteristics are developed by the metal-based nanoparticles that differ from the bulk material. Metallic NPs affect the metabolic activity of microbial communities with high affinity for cellular penetration causing membrane damage (Wang et al. 2017). Hence, shifting the focus to the antibacterial and antibiofilm activity of nanoantibiotics may reveal new antibiofilm preparations, and lead to significant improvement of microbial eradication.

To some extent, some factors affecting the biosynthesis of Ag- and Cu-NPs were optimized and data revealed that the optimum Ag-NPs synthesis was achieved at 24 h contact time between biomass filtrate and the precursor salt. The concentration of 5 mM AgNO3 was utilized like other reports (Bukhari et al. 2021; Tiwari et al. 2016).

On the other hand, the maximum production and stability of Cu-NPs was fulfilled after 48 h, using 5 mM CuSO4 which is considered the most widely used copper salt for nano-synthesis (Hassan et al. 2019) (Bukhari et al. 2021). For both NPs, alkaline pH was utilized to optimize the reaction conditions that resulted in maximum yield production and also help in controlling the shape, size and stability of nano-metals (Khalil et al. 2022).

In this study, *Streptomyces* isolate 29 was used for the biological synthesis of Ag-NPs, and Cu-NPs. Biogenic Ag-NPs were synthesized within 24 h with a brown color **(**Fig. 1a), and UV/Vis spectra at 478 nm (Fig. 1c). The color change could be attributed to the reduction of Ag^+^ by metabolites present in the cell‐free supernatant of *Streptomyces* isolate S29. Production of Ag-NPs by other bacteria and *Streptomyces* was also verified by the formation of brown color and by the presence of absorption spectra at 420–450 nm (Chauhan et al. 2013; Saravana Kumar et al. 2015; Soltaninezhad et al. 2015; Zonooz and Salouti 2011).

Biologically formed Cu-NPs were characterized by blue color (Fig. 1b), as reported by Shobha and coauthors (Shobha et al. 2014), with a maximum absorption peak at 594 nm (Fig. 1d). The observed spectrum revealed that the characteristic pattern of Cu-NPs was accomplished by reducing copper ions with metabolites in cell-free supernatants. At the same instance, UV/visible spectroscopy analysis of Cu-NPs fabricated by endophytic *actinomycetes* exhibited a peak at 600 nm (Saad et al. 2018). This variation in spectral data depends on the difference in the reduction reaction and capping nature (Balraj et al. 2017; Rajivgandhi et al. 2022).

The zeta potentials of the biosynthesized Ag-NPs, and Cu-NPs were, -29.7, and -33.7 mv, respectively. The formed nanoparticles were negatively charged, which could be attributed to the type of capped amino acid in the culture biomass during metallic reduction. Furthermore, high zeta potential values indicated the presence of a high repulsive force between the particles**;** hence, no agglomerates were formed by the synthesized nanoparticles. In the same instance, *Zingiber officinale* synthesized stable Cu-NPs with a zeta potential − 44.3 mv (Pandit et al. 2017). Additionally, the PDI ranged from 0.1 to 0.546, indicating homogeneity of the formed nanoparticles, and monodispersed particles with few aggregates.

The same data were confirmed by TEM/SAED analysis which indicated monodispersed spherical crystalline Ag-NPs with a nanodiameter of 10–20 nm (Fig. 2a). Elemental production of Ag nanometals was also confirmed by EDX analysis (Fig. 2c) and the presence of other elements indicates the biogenic capping of the Ag metal. FTIR also showed the presence of functional groups with the appearance of band spectra at 1057 and 1235 cm^−1^ (Fig. 2e) indicating protein capping which plays an important role in the stability of Ag-NPs (Ganachari et al. 2012; Karthik et al. 2020). The bioreduction of silver ions into Ag-NPs has been reported previously by different bacterial, *Streptomyces* and fungal species with spherical crystalline structures (Mabrouk et al. 2021). *Streptomyces* produce different natural products such as alkaloids, flavonoids, lipids, polysaccharides, enzymes and metabolites that assist in the biosynthesis and capping of the produced Ag-NPs (Chauhan et al. 2013; Singh et al. 2014). Different enzymes are also involved in this process (Ovais et al. 2018) such as NADPH-dependent nitrate reductase, which is induced by nitrate ions and reduces silver ions to metallic silver (Kalishwaralal et al. 2008).

The biosynthesized Cu-NPs had a monodispersed spherical to square and hexagonal particles with particle sizes of 25–35 nm (Fig. 2b). Similarly, *Shewanella oneidensis* produced spherical Cu-NPs with a particle size range of 20–50 nm (Salem and Fouda 2021). The biologically synthesized Cu-NPs obtained from the culture supernatant of endophytic *Streptomyces* sp. revealed a particle size of 80 nm (Husein et al. 2019). Elemental signals of Cu-NPs were detected by EDX analysis (Fig. 2d). The EDX signals of Cu were more distinct compared to other signals and they were similar to EDX signals of biogenic Cu-NPs reduced by ascorbic acid in aqueous CTAB solution (Biçer and Şişman 2010). The EDX signals of C developed from biological capping, reduction and formation of biogenic Cu-NPs. The FTIR spectra of Cu-NPs showed the presence of different functional groups like alcohol, carboxyl, alkane, and alkene groups, which play a very significant role in biocapping Cu-NPs (Durán et al. 2011; Joseph et al. 2016; Mahmoudvand et al. 2020) (Fig. 2f).

Sequencing and phylogenetic analysis of the 16S rRNA gene of the S29 isolate showed high similarity (98.82%) to *Streptomyces thinghirensis* (Fig. 3a). Also, S29 is characterized by spiral spore chains with a smooth surface (Fig. 3b) with similar morphological characteristics to *Streptomyces thinghirensis* isolated from Thinghir*,* Morocco (Loqman et al. 2022). *Streptomyces thinghirensis* has been previously isolated from rhizosphere soil (Loqman et al. 2009) and from Qassim (Rehan et al. 2023)*.* Predictive analysis of secondary metabolites produced by *S. thinghirensis* indicates the potential production of terpenes, butyrolactone, siderophore, lantipeptide, polyketide synthase, non-ribosomal peptide synthetase cluster, and others (Rehan et al. 2023) that could contribute to the bioreduction and capping of Ag-NPs and Cu-NPs.

Furthermore, the present study evaluated the antimicrobial and antibiofilm efficacy of the biologically prepared nanoparticles. Ag-NPs exhibited prominent antimicrobial and bactericidal activity against all tested isolates (Table 1). Likewise, the biologically prepared Ag-NPs using *Streptomyces hirsutus* strain SNPGA-8 (Pallavi et al. 2022), *Streptomyces enissocaesilis* (Shaaban and El‐Mahdy 2018), and *Streptomyces griseorubens* AU2 (Baygar and Ugur 2017) and (Sadhasivam et al. 2010) were effective against MDR pathogens. The antimicrobial activity of Ag-NPs could be attributed to their small partial size in the nanorange (1–100 nm), with rapid and efficient cellular adhesion, membrane penetration, and high cellular interaction (Morones et al. 2005). In addition, Ag-NPs exhibit various antimicrobial efficacies, including destruction of the cell membrane, enhanced cellular penetration, and high affinity in binding cellular components with inhibition of protein synthesis, DNA replication, and alteration of the respiratory chain (Baptista et al. 2018; Cheeseman et al. 2020). Moreover, Ag-NPs demonstrated a significant decrease in bacterial attachment and biofilm formation (*P* < 0.001, Figs. 4–5) and distributed mature biofilm (Figs. 6–7)*.* This could be attributed to the ability of Ag-NPs to neutralize the adhesive elements that initiate biofilm formation, with further elimination of bacterial adhesion, and prevention of biofilm formation (Goel et al. 2021; More et al. 2023; Rai et al. 2012). Ag-NPs disrupt cellular permeability, and lipid bilayer integrity, increase membrane internalization and cause cells to rupture. Their small particle size assisted their internalization and diffusion through the biofilm matrix with disruption of membrane integrity and morphological changes in cell shape and arrangement (Gurunathan et al. 2014; Qing et al. 2018). At the same instance, Ag-NPs generate reactive oxygen species that interact with cellular components, disrupt vital organs, respiratory enzymes and metabolic processes and lead to cell death. In addition, Ag-NPs inhibited the quorum sensing signaling mechanisms of *P. aeruginosa* and related virulence factors. Also, the production of exopolysaccharide and biofilm formation by *P. aeruginosa* were significantly eliminated (Saeki et al. 2021). Previous studies elicited the antibiofilm activity of the biosynthesized nanoparticles in a concentration dependent manner, Ag-NPs (0.1- 1.0 μg/mL) synthesized by extracts of *Allophylus cobbe* leaves eliminated mature biofilm of both Gram-negative pathogens (*P. aeruginosa,* and* S. flexneri)* and Gram-positive pathogens *(S. aureus* and* S. pneumoniae*) (Zaki and Husain 2016).

In our study, all the tested standard and clinical isolates responded differently to the prepared Cu-NPs since no MIC or MBC values were detected upon treatment of bacterial cultures with Cu-NPs (Table 1). Compared to Ag-NPs; the lower toxicity of Cu-NPs was due to, the essentiality of Cu in many cellular physiological systems and its role as a cofactor for bacterial respiration enzymes including cytochrome C oxidase and NADH dehydrogenase. Hence, treatment of bacterial cultures with 5 mM CuSO_4_ appeared to be bacteriostatic not bactericidal. Consequently, Cu-NPs act as a fundamental source of copper required for cell growth rather than killing (Luong et al. 2022).

Moreover, the biogenic Cu-NPs also exhibited antibiofilm activity with elimination of bacterial attachment disruption of mature biofilm (Table 2**, **Fig. 4–7). Also, the loss of cell viability within the biofilm matrix was also detected by TTC assay. This could also be attributed to the low particle size of Cu-NPs with high penetration ability associated with internal protein binding and cellular damage. Cu-NPs have highly active redox potential and can generate reactive oxygen species (ROS), such as peroxide free radicals, which cause oxidative stress and subsequent damage to cellular components such as lipids, proteins, and nucleic acids (Karlsson et al. 2013; VanWinkle et al. 2009). Cu-NPs interact with cell membranes, affect bacterial integrity and disrupt selective metal ion permeability (Sharpe et al. 2011). The antimicrobial activity of Cu-NPs prepared using extracts of *Opuntia ficusindica* and *Geranium* showed an antimicrobial activity in a particle size dependent manner.

At the molecular level, *carO* and *bssS* genes encode small cytoplasmic proteins that significantly regulate biofilm formation in *A. baumannii* and *K. pneumoniae,* respectively (Cabral et al. 2011; Domka et al. 2006). In *P. aeruginosa*, the *pelA* gene is involved in the synthesis and transport of polysaccharides (Colvin et al. 2012). Both Ag-NPs and Cu-NPs significantly eliminated the expression of biofilm-associated genes (Fig. 8). Similarly, Ag-NPs reduced the expression of the genes *fnbA* and *fnbB* involved in biofilm formation (Gheidar et al. 2018), in addition to the *ica A* and *ica D* genes (Pourmbarak Mahnaie and Mahmoudi 2020). Also, Ag-NPs significantly suppressed the expression of virulence and biofilm regulatory genes (*fimH, rmpA,* and *mrkA*) in MDR *K. pneumoniae* isolates (Mousavi et al. 2023). This could be attributed to the prominent penetration of nanometals into the bacterial cells, binding with cytoplasmic proteins and interruption of cellular functions including gene expression (biofilm-regulatory genes).

In conclusion, *Streptomyces* isolates were purified from soil with the ability to survive in high metallic concentrations and were utilized for the biosynthesis of nanometals. The effect of the synthesized nanometals on the biofilm formation by MDR isolates showed a prominent effect. Furthermore, the influence of Ag-NPs and Cu-NPs on mature biofilm revealed significant eradication of the biofilm matrix with a marked decrease in viable cells as detected by TTC assay. Through this research, we were able to synthesize and characterize nanometals, and study their abilities to control biofilm formation and bacterial cells inside matrix polysaccharides. This study provides a prospective strategy and innovative approach for novel antimicrobial and antibiofilm agents.

### Supplementary Information

**Additional file 1: Table S1**. Primer sets used for RT-PCR. **Table S2**. UV/Vis spectroscopy data of the cell-free supernatant of *Streptomyces *isolate S29 either untreated or treated with AgNO3 and CuSO4 (5 mM each), in a wavelength range of 300–1000 nm. **Figure S1**: Optimization of NPs through (a) different concentrations (3 and 5 mM) of CuSO4, (b) different salts of CuSO4 and CuCL2 at fixed concentration (5 mM) with particle size and zeta potential of CuCL2 and different incubation contact time (24 h and 48 h) of (d) Ag-NPs and (e) Cu-NPs. **Figure S2**: Particle size distribution of (a) Ag-NPs, and (b) Cu-NPs and zeta potentials of biosynthesized (c) Ag-NPs, and (d) Cu-NPs. **Figure S3**; Effect of different concentration of Ag-NPs and Cu-NPs on the initial attachment and biofilm formation; a) untreated biofilm of *A. baumannii *ATCC 19606, and *A. baumannii *A11 stained with crystal violet (CV) method and biofilm treated with (b) Ag-NPs (2x, 1x, 0.5x and 0.25x MIC) and (c) Cu-NPs (50% and 25% concentration) stained with CV. d) untreated biofilm of *A. baumannii *ATCC 19606, and *A. baumannii *A11 stained with Tri-phenyl tetrazolium chloride (TTC) method and treated biofilm with (e) Ag-NPs (2x, 1x, 0.5x and 0.25x MIC) and (f) Cu-NPs (50% and 25% concentration) stained with TTC. **Figure S4**; Effect of different concentration of Ag-NPs and Cu-NPs on the initial attachment and biofilm formation; a) untreated biofilm of *K. pneumoniae *ATCC 51503, and *K. pneumoniae *K7 stained with crystal violet (CV) method and biofilm treated with (b) Ag-NPs (2x, 1x, 0.5x and 0.25x MIC) and (c) Cu-NPs (50% and 25% concentration) stained with CV. d) untreated biofilm of *K. pneumoniae *ATCC 51503, and *K. pneumoniae *K7 stained with Tri-phenyl tetrazolium chloride (TTC) method and treated biofilm with (e) Ag-NPs (2x, 1x, 0.5x and 0.25x MIC) and (f) Cu-NPs (50% and 25% concentration) stained with TTC. **Figure S5**; Effect of different concentration of Ag-NPs and Cu-NPs on the initial attachment and biofilm formation; a) untreated biofilm of *P. aeruginosa *PAO1 and *P. aeruginosa *P2 stained with crystal violet (CV) method and biofilm treated with (b) Ag-NPs (2x, 1x, 0.5x and 0.25x MIC) and (c) Cu-NPs (50% and 25% concentration) stained with CV. d) untreated biofilm of *P. aeruginosa *PAO1 and *P. aeruginosa *P2 stained with Tri-phenyl tetrazolium chloride (TTC) method and treated biofilm with (e) Ag-NPs (2x, 1x, 0.5x and 0.25x MIC) and (f) Cu-NPs (50% and 25% concentration) stained with TTC. **Figure S6**; Effect of different concentration of Ag-NPs and Cu-NPs on mature biofilm; a) untreated biofilm of *A. baumannii *ATCC 19606, and *A. baumannii *A11 stained with crystal violet (CV) method and biofilm treated with (b) Ag-NPs (16x, 8x, 4x, 2x, 1x, 0.5x and 0.25x MIC) and (c) Cu-NPs (100%, 50% and 25%) stained with CV method. d) untreated biofilm of *A. baumannii *ATCC 19606, and *A. baumannii *A11 stained with Tri-phenyl tetrazolium chloride (TTC) method and treated biofilm with (e) Ag-NPs (16x, 8x, 4x, 2x, 1x, 0.5x and 0.25x MIC) and (f) Cu-NPs (100%, 50% and 25% concentration) stained with TTC. **Figure S7**; Effect of different concentration of Ag-NPs and Cu-NPs on mature biofilm; a) untreated biofilm of *K. pneumoniae *ATCC 51503, and *K. pneumoniae *K7 stained with crystal violet (CV) method and biofilm treated with (b) Ag-NPs (16x, 8x, 4x, 2x, 1x, 0.5x and 0.25x MIC) and (c) Cu-NPs (100%, 50% and 25% concentration) stained with CV. d) untreated biofilm of *K. pneumoniae *ATCC 51503, and *K. pneumoniae *K7 stained with Tri-phenyl tetrazolium chloride (TTC) method and treated biofilm with (e) Ag-NPs (16x, 8x, 4x, 2x, 1x, 0.5x and 0.25x MIC) and (f) Cu-NPs (100, 50% and 25 % concentration) stained with TTC. **Figure S8**; Effect of different concentration of Ag-NPs and Cu-NPs on the mature; a) untreated biofilm of *P. aeruginosa *PAO1 and *P. aeruginosa *P2 stained with crystal violet (CV) method and biofilm treated with (b) Ag-NPs (16x, 8x, 4x, 2x, 1x, 0.5x and 0.25x MIC and (c) Cu-NPs (100%, 50% and 25% concentration) stained with CV method. d) untreated biofilm of *P. aeruginosa *PAO1 and *P. aeruginosa *P2 stained with Tri-phenyl tetrazolium chloride (TTC) method and treated biofilm with (e) Ag-NPs (16x, 8x, 4x, 2x, 1x, 0.5x and 0.25x MIC) and (f) Cu-NPs (100%, 50% and 25% concentration) stained with TTC.

## Data Availability

All data generated or analyzed during this study are included in this published article and its Additional files.

## Abbreviations

CV: Crystal violet
EDX: Analysis and energy dispersive X-ray diffraction
MDR: Multidrug resistant
MIC: Minimal inhibitory concentration
MBC: Minimal bactericidal concentration
NPs: Nanoparticles
SAED: Selected area electron diffraction pattern
TSB: Tryptic soy broth
TTC: 2,3,5 Tri-phenyl tetrazolium chloride
TEM: Transmission electron microscopy
XDR: Extensively drug-resistant
