# Supplementary material for: Antibiofilm activity of biosynthesized silver and copper nanoparticles using Streptomyces S29
Source: AMB Express. 2023 Dec 6;13:139. doi: 10.1186/s13568-023-01647-3 (PMC10700280; doi:10.1186/s13568-023-01647-3)
Supplement: Supplementary file 1 — Additional file 1: Table S1. Primer sets used for RT-PCR. Table S2. UV/Vis spectroscopy data of the cell-free supernatant of Streptomyces isolate S29 either untreated or treated with AgNO3 and CuSO4 (5 mM each), in a wavelength range of 300–1000 nm. Figure S1: Optimization of NPs through (a) different concentrations (3 and 5 mM) of CuSO4, (b) different salts of CuSO4 and CuCL2 at fixed concentration (5 mM) with particle size and zeta potential of CuCL2 and different incubation contact time (24 h and 48 h) of (d) Ag-NPs and (e) Cu-NPs. Figure S2: Particle size distribution of (a) Ag-NPs, and (b) Cu-NPs and zeta potentials of biosynthesized (c) Ag-NPs, and (d) Cu-NPs. Figure S3; Effect of different concentration of Ag-NPs and Cu-NPs on the initial attachment and biofilm formation; a) untreated biofilm of A. baumannii ATCC 19606, and A. baumannii A11 stained with crystal violet (CV) method and biofilm treated with (b) Ag-NPs (2x, 1x, 0.5x and 0.25x MIC) and (c) Cu-NPs (50% and 25% concentration) stained with CV. d) untreated biofilm of A. baumannii ATCC 19606, and A. baumannii A11 stained with Tri-phenyl tetrazolium chloride (TTC) method and treated biofilm with (e) Ag-NPs (2x, 1x, 0.5x and 0.25x MIC) and (f) Cu-NPs (50% and 25% concentration) stained with TTC. Figure S4; Effect of different concentration of Ag-NPs and Cu-NPs on the initial attachment and biofilm formation; a) untreated biofilm of K. pneumoniae ATCC 51503, and K. pneumoniae K7 stained with crystal violet (CV) method and biofilm treated with (b) Ag-NPs (2x, 1x, 0.5x and 0.25x MIC) and (c) Cu-NPs (50% and 25% concentration) stained with CV. d) untreated biofilm of K. pneumoniae ATCC 51503, and K. pneumoniae K7 stained with Tri-phenyl tetrazolium chloride (TTC) method and treated biofilm with (e) Ag-NPs (2x, 1x, 0.5x and 0.25x MIC) and (f) Cu-NPs (50% and 25% concentration) stained with TTC. Figure S5; Effect of different concentration of Ag-NPs and Cu-NPs on the initial attachment and biofilm formation; [file 13568_2023_1647_MOESM1_ESM.pdf]

Antibiofilm activity of Biosynthesized Silver and Copper Nanoparticles Using *Streptomyces* S29

Soha Lotfy Elshaer* and Mona I. Shaaban *

Department of Microbiology and Immunology, Faculty of Pharmacy, Mansoura University, Mansoura 35516, Egypt

* Correspondence: mona_ibrahem@mans.edu.eg; dr_sohaloftyeldamarawy@mans.edu.eg

Table S1

table 1. Primer sets used for RT-PCR

Organism	Primer	Sequence (5′→3′)	AT (°C)	Amplicon size (bp)
A. baumannii	CarO F	TAGTAAGCGCCTACTTCACC	55	207
	CarO R	CAGGTGCGGCGTATTTAGAT		
	RpoB Ac F	ACAAAGTAATGCGTCCAGGC	57	121
	RpoB Ac R	CGGTTGAACTTCATACGACCT		
K. pneumonia	BssS F	GATTCAATTTTGGCGATTCCTGC	60	225
	BssS R	TAATGAAGTCATTCAGACTCATCC		
	RpoD F	AAGACGAAGATGAAGACGCC	57	129
	RpoD R	CTTTGGCTTTGATGGTGTCG		
P. aeruginosa	PelA F	AAGAACGGATGGCTGAAGG	58	148
	PelA R	TTCCTCACCTCGGTCTCG		
	RpoD F	CGAACTGCTTGCCGACTT	56	131
	RpoD R	GCGAGAGCCTCAAGGATAC		

AT; Annealing Temperature, F; Forward, R; Reverse; bp; base pair

Table S2

table 2. UV/Vis spectroscopy data of the cell-free supernatant of *Streptomyces* isolate S29 either untreated or treated with AgNO3 and CuSO4 (5 mM each), in a wavelength range of 300–1000 nm.

**NPs**	**Time of production**	***Streptomyces* S29 Cell free Supernatant**		***Streptomyces* S29 Treated with salt**	
		**λ_{max}**	**A (nm)**	**λ_{max}**	**A (nm)**
**Ag-NPs**	**24 h**	**400**	**0.459**	**478**	**1.517**
**Ag-NPs**	**48 h**	**400**	**0.4**	**478**	**1.4**
**Cu-NPs**	**24 h**	**669**	**0.2**	**627**	**0.52**
**Cu-NPs**	**48 h**	**673**	**0.237**	**594**	**0.565**

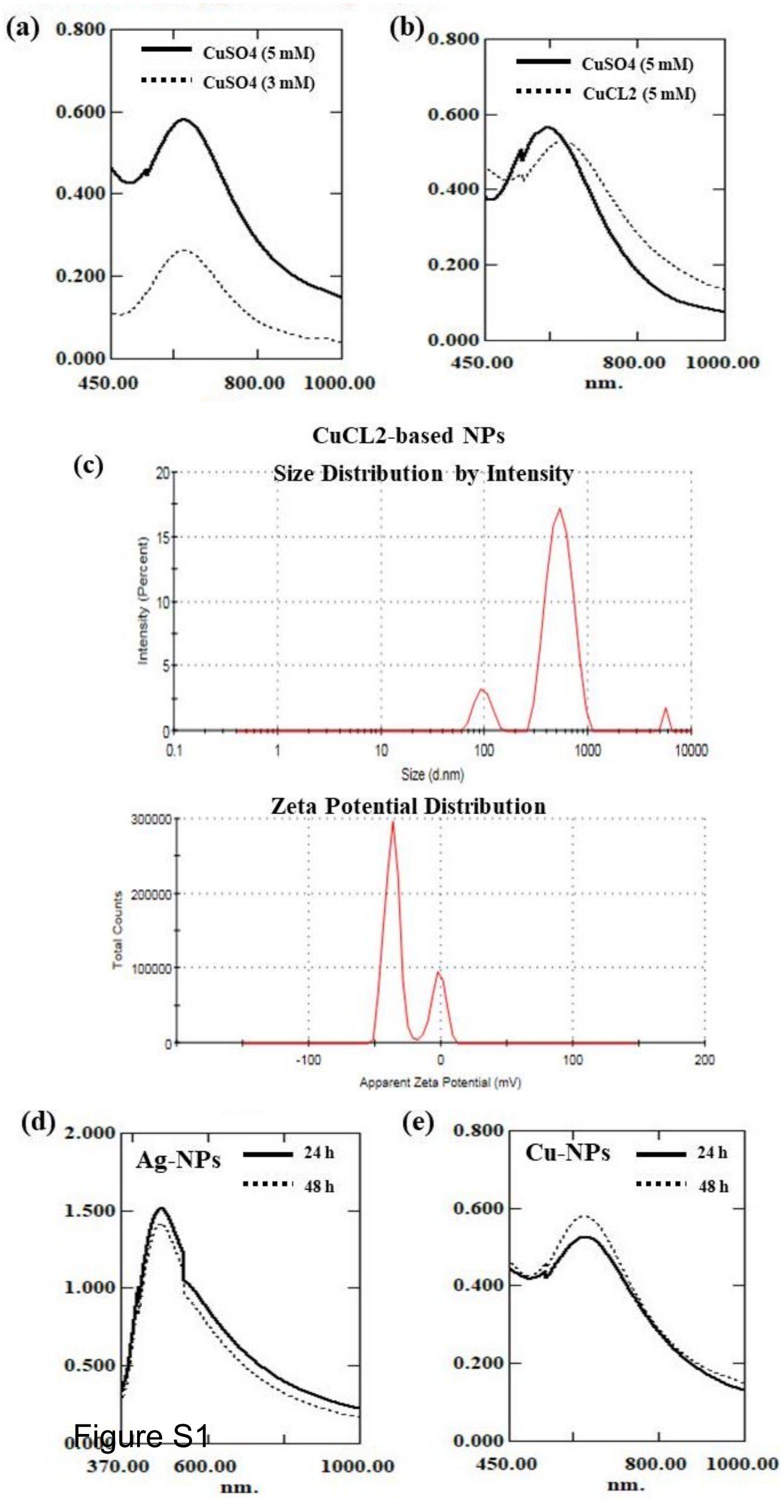

figure 1: Optimization of NPs through (a) different concentrations (3 and 5 mM) of CuSO4, (b) different salts of CuSO4 and CuCL2 at fixed concentration (5 mM) with particle size and zeta potential of CuCL2 and different incubation contact time (24 h and 48 h) of (d) Ag-NPs and (e) Cu-NPs

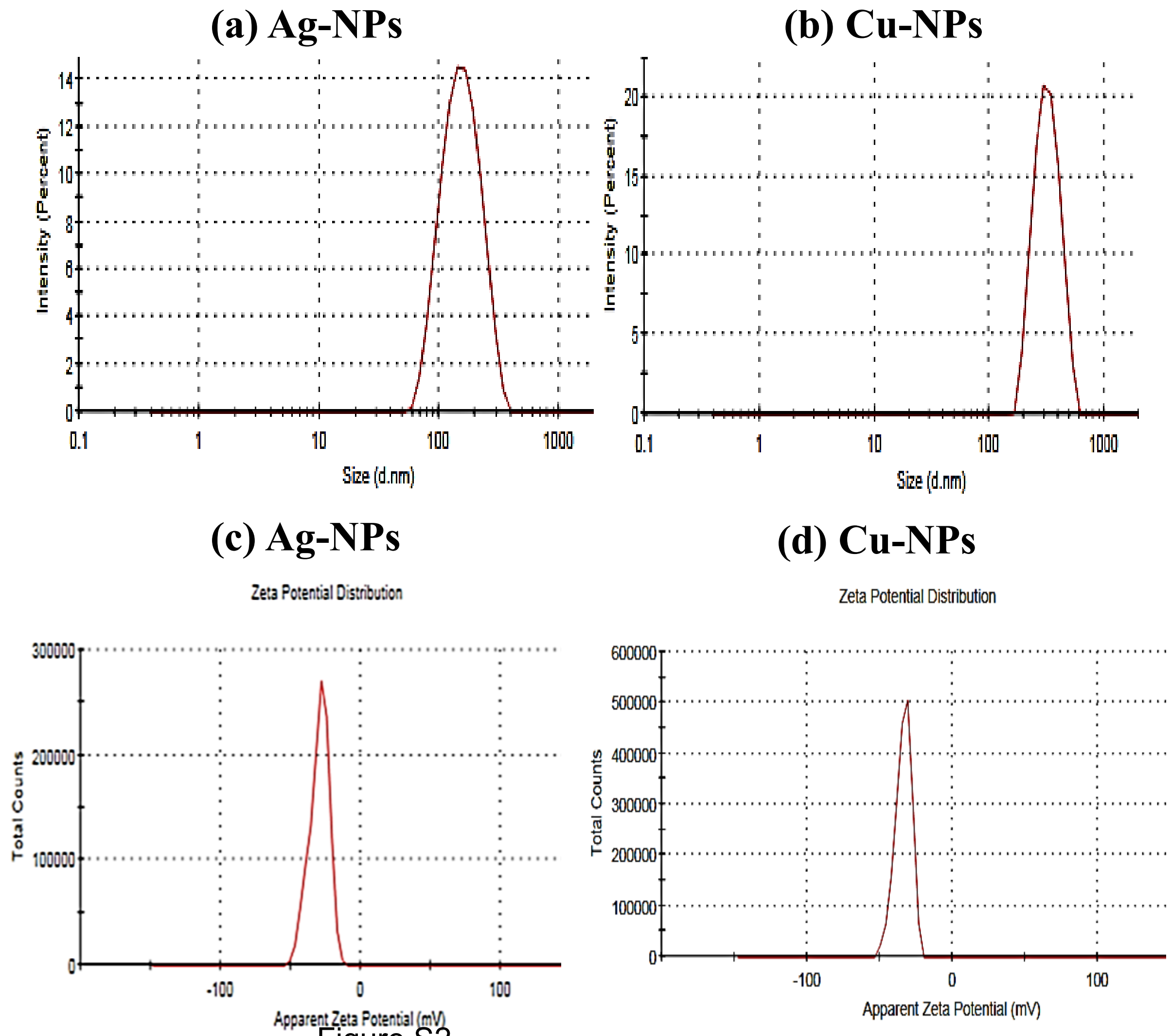

Figure S2

figure 2: Particle size distribution of (a) Ag-NPs, and (b) Cu-NPs and zeta potentials of biosynthesized (c) Ag-NPs, and (d) Cu-NPs

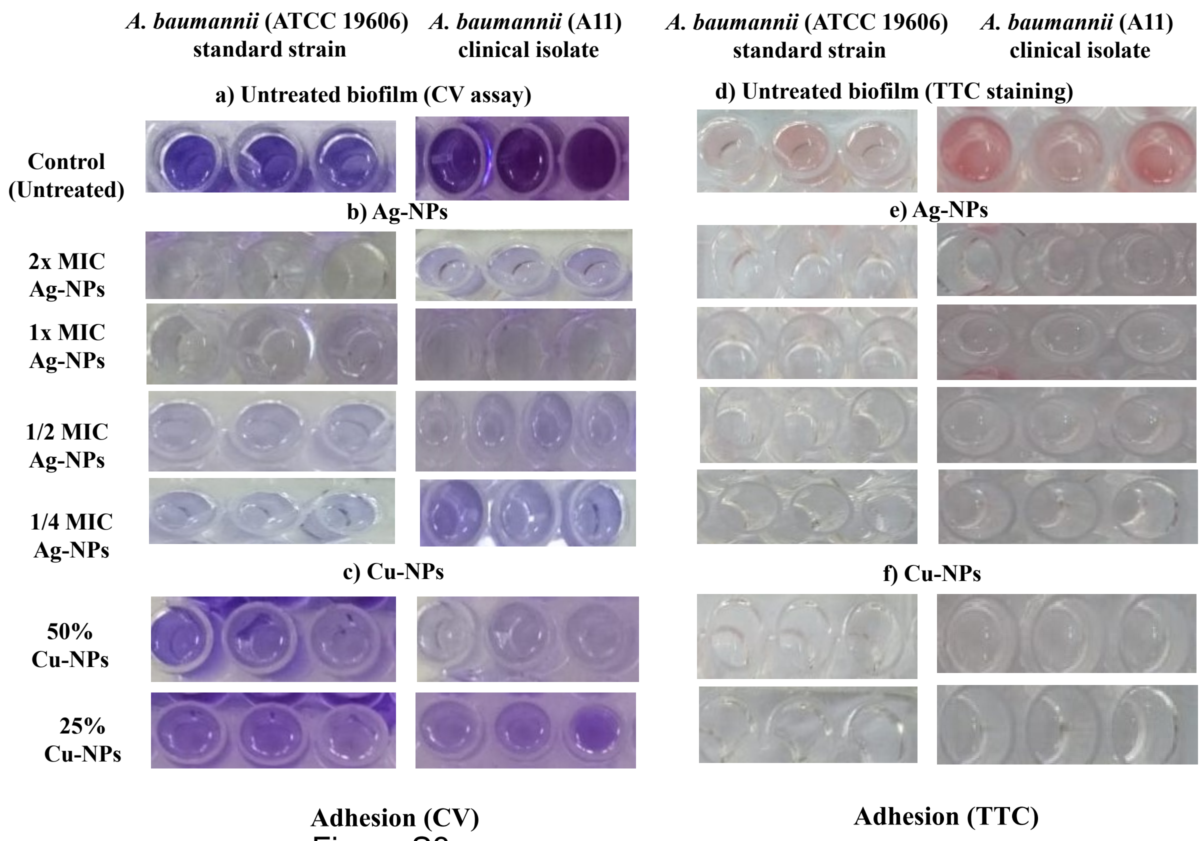

Figure S3

figure 3; Effect of different concentration of Ag-NPs and Cu-NPs on the initial attachment and biofilm formation; a) untreated biofilm of *A. baumannii* ATCC 19606, and *A. baumannii* A11 stained with crystal violet (CV) method and biofilm treated with (b) Ag-NPs (2x, 1x, 0.5x and 0.25x MIC) and (c) Cu-NPs (50% and 25% concentration) stained with CV. d) untreated biofilm of *A. baumannii* ATCC 19606, and *A. baumannii* A11 stained with Tri-phenyl tetrazolium chloride (TTC) method and treated biofilm with (e) Ag-NPs (2x, 1x, 0.5x and 0.25x MIC) and (f) Cu-NPs (50% and 25% concentration) stained with TTC.

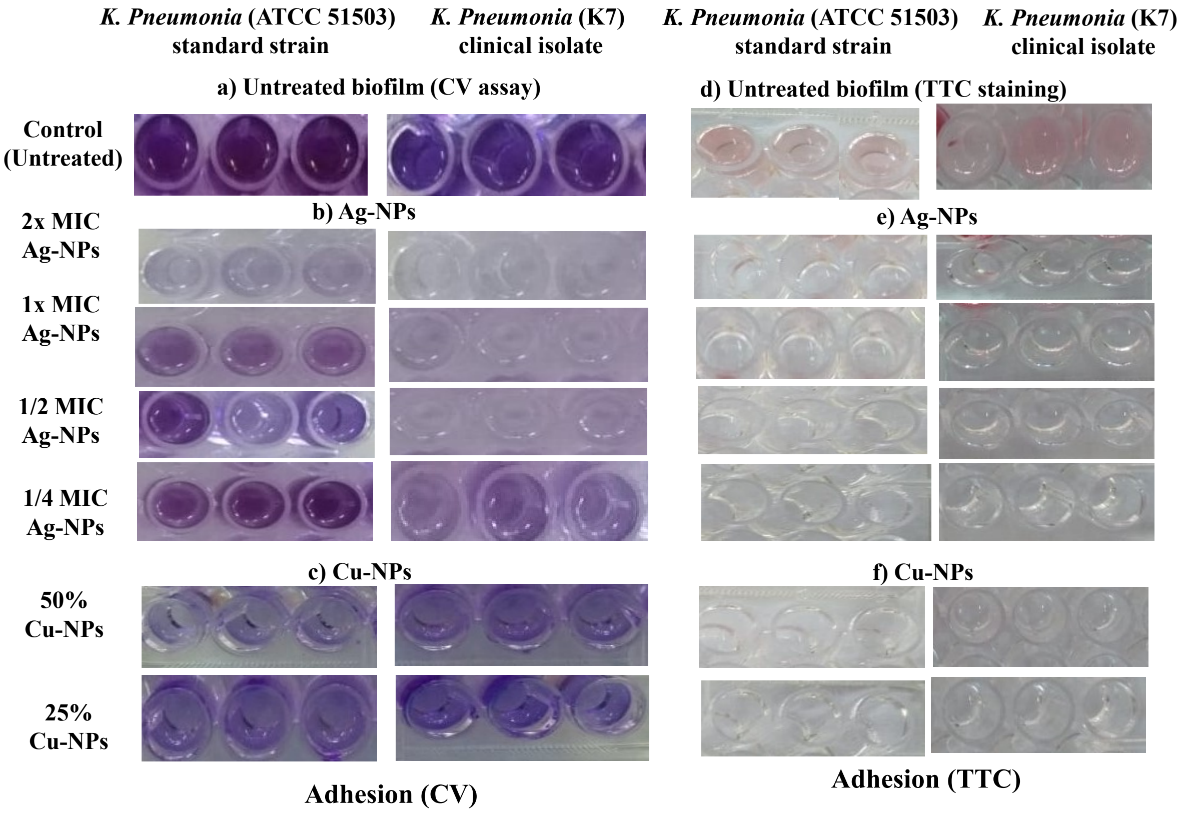

Figure S4

figure 4; Effect of different concentration of Ag-NPs and Cu-NPs on the initial attachment and biofilm formation; a) untreated biofilm of *K. pneumoniae* ATCC 51503, and *K. pneumoniae* K7 stained with crystal violet (CV) method and biofilm treated with (b) Ag-NPs (2x, 1x, 0.5x and 0.25x MIC) and (c) Cu-NPs (50% and 25% concentration) stained with CV. d) untreated biofilm of *K. pneumoniae* ATCC 51503, and *K. pneumoniae* K7 stained with Tri-phenyl tetrazolium chloride (TTC) method and treated biofilm with (e) Ag-NPs (2x, 1x, 0.5x and 0.25x MIC) and (f) Cu-NPs (50% and 25% concentration) stained with TTC.

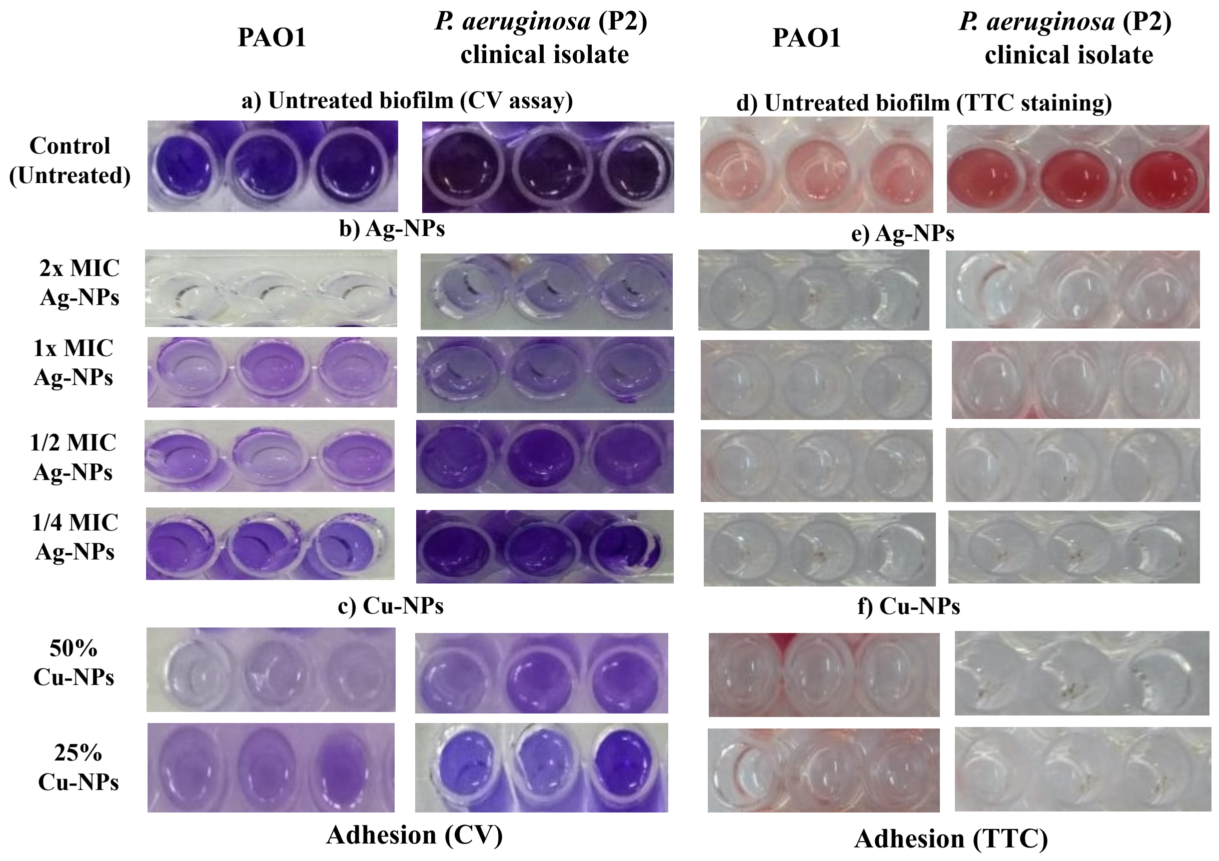

Figure S5

figure 5; Effect of different concentration of Ag-NPs and Cu-NPs on the initial attachment and biofilm formation; a) untreated biofilm of *P. aeruginosa* PAO1 and *P. aeruginosa* P2 stained with crystal violet (CV) method and biofilm treated with (b) Ag-NPs (2x, 1x, 0.5x and 0.25x MIC) and (c) Cu-NPs (50% and 25% concentration) stained with CV. d) untreated biofilm of *P. aeruginosa* PAO1 and *P. aeruginosa* P2 stained with Tri-phenyl tetrazolium chloride (TTC) method and treated biofilm with (e) Ag-NPs (2x, 1x, 0.5x and 0.25x MIC) and (f) Cu-NPs (50% and 25% concentration) stained with TTC.

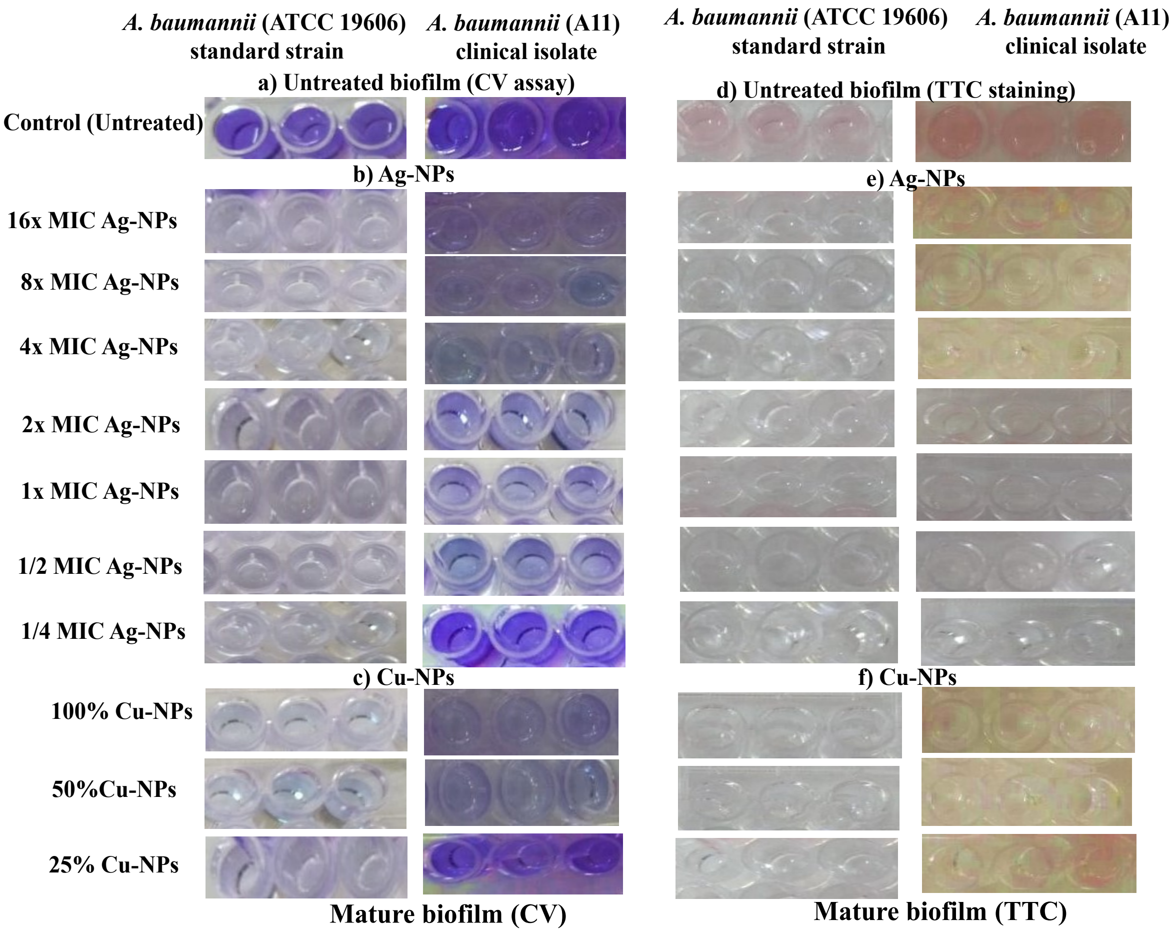

Figure S6

figure 6; Effect of different concentration of Ag-NPs and Cu-NPs on mature biofilm; a) untreated biofilm of *A. baumannii* ATCC 19606, and *A. baumannii* A11 stained with crystal violet (CV) method and biofilm treated with (b) Ag-NPs (16x, 8x, 4x, 2x, 1x, 0.5x and 0.25x MIC) and (c) Cu-NPs (100%, 50% and 25%) stained with CV method. d) untreated biofilm of *A. baumannii* ATCC 19606, and *A. baumannii* A11 stained with Tri-phenyl tetrazolium chloride (TTC) method and treated biofilm with (e) Ag-NPs (16x, 8x, 4x, 2x, 1x, 0.5x and 0.25x MIC) and (f) Cu-NPs (100%, 50% and 25% concentration) stained with TTC.

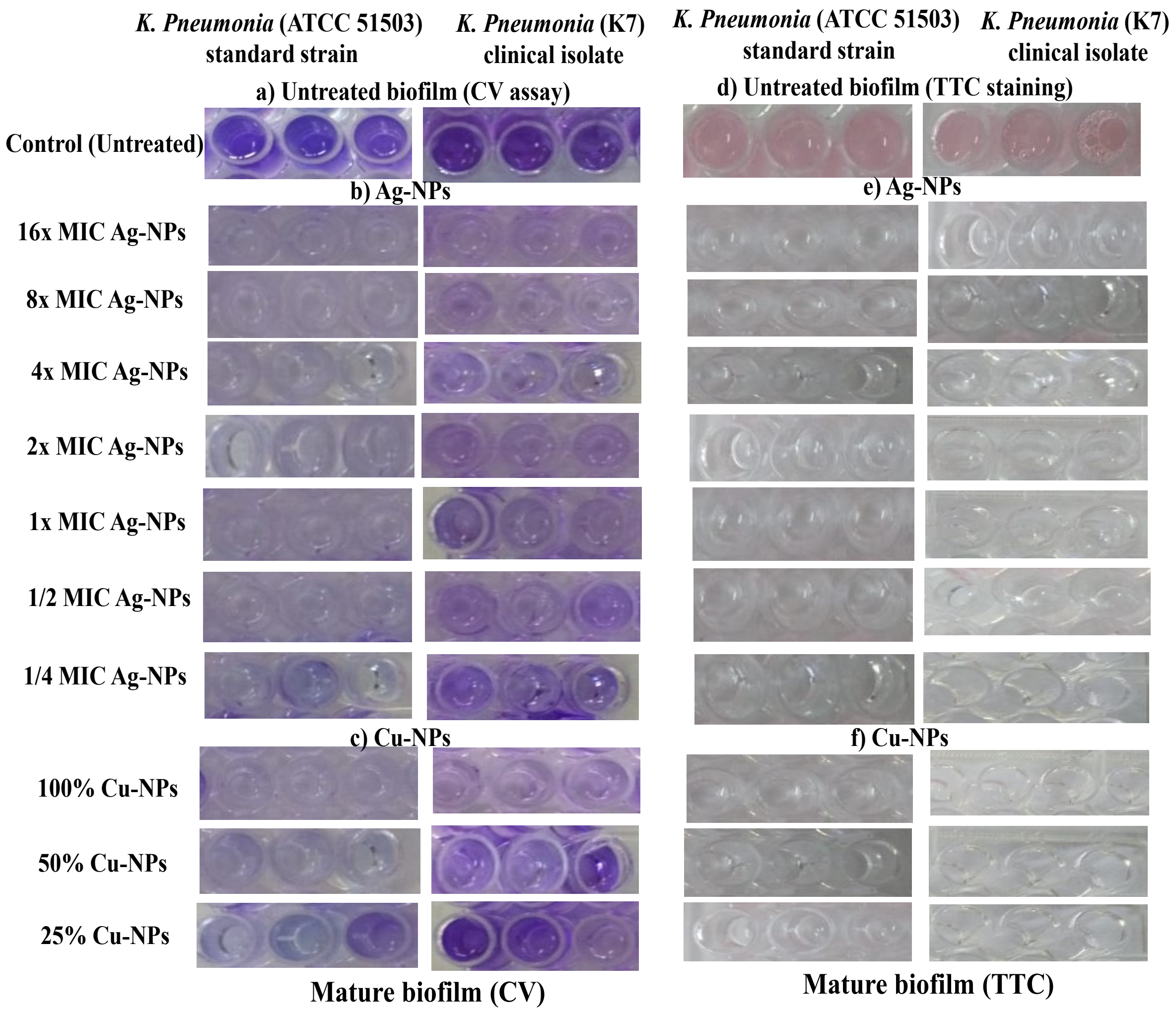

Figure S7

figure 7; Effect of different concentration of Ag-NPs and Cu-NPs on mature biofilm; a) untreated biofilm of *K. pneumoniae* ATCC 51503, and *K. pneumoniae* K7 stained with crystal violet (CV) method and biofilm treated with (b) Ag-NPs (16x, 8x, 4x, 2x, 1x, 0.5x and 0.25x MIC) and (c) Cu-NPs (100%, 50% and 25% concentration) stained with CV. d) untreated biofilm of *K. pneumoniae* ATCC 51503, and *K. pneumoniae* K7 stained with Tri-phenyl tetrazolium chloride (TTC) method and treated biofilm with (e) Ag-NPs (16x, 8x, 4x, 2x, 1x, 0.5x and 0.25x MIC) and (f) Cu-NPs (100, 50% and 25 % concentration) stained with TTC.

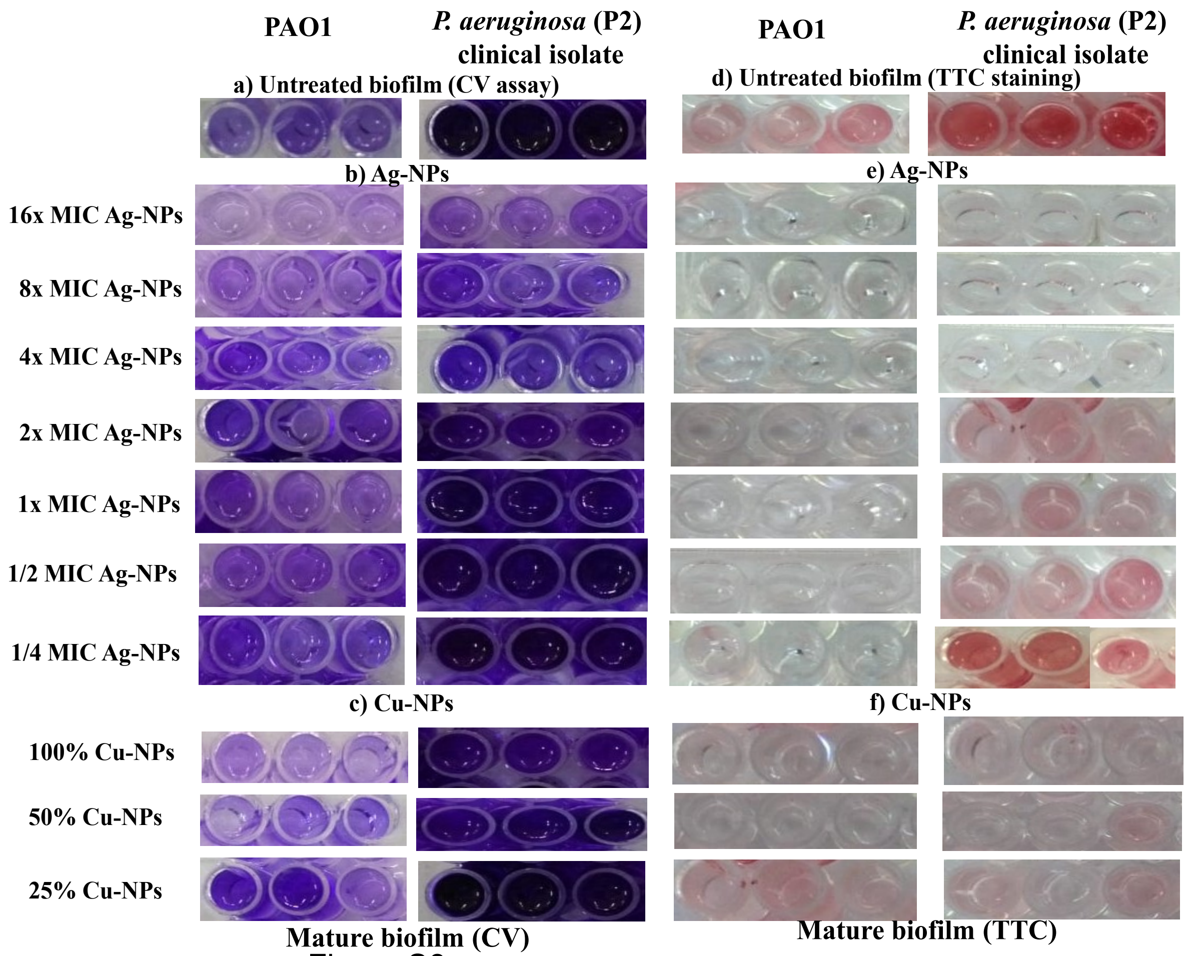

Figure S8

figure 8; Effect of different concentration of Ag-NPs and Cu-NPs on the mature; a) untreated biofilm of *P. aeruginosa* PAO1 and *P. aeruginosa* P2 stained with crystal violet (CV) method and biofilm treated with (b) Ag-NPs (16x, 8x, 4x, 2x, 1x, 0.5x and 0.25x MIC and (c) Cu-NPs (100%, 50% and 25% concentration) stained with CV method. d) untreated biofilm of *P. aeruginosa* PAO1 and *P. aeruginosa* P2 stained with Tri-phenyl tetrazolium chloride (TTC) method and treated biofilm with (e) Ag-NPs (16x, 8x, 4x, 2x, 1x, 0.5x and 0.25x MIC) and (f) Cu-NPs (100%, 50% and 25% concentration) stained with TTC.
